# Owls May Use Faeces and Prey Feathers to Signal Current Reproduction

**DOI:** 10.1371/journal.pone.0003014

**Published:** 2008-08-20

**Authors:** Vincenzo Penteriani, Maria del Mar Delgado

**Affiliations:** Department of Conservation Biology, Estación Biológica de Doñana, CSIC, Seville, Spain; Stanford University, United States of America

## Abstract

**Background:**

Many animals communicate by marking focal elements of their home range with different kinds of materials. Visual signaling has been demonstrated to play a previously unrecognized role in the intraspecific communication of eagle owls (*Bubo bubo*), in both territorial and parent-offspring contexts. Visual signals may play a role in a variety of circumstances in this crepuscular and nocturnal species.

**Methodology/Principal Findings:**

Here, we report that a large amount of extremely visible white faeces and prey feathers appear during the breeding season on posts and plucking sites in proximity to the nest, potentially representing a way for eagle owls to mark their territory. We present descriptive and experimental evidence showing that faeces and prey remains could act as previously unrecognized visual signals in a nocturnal avian predator. This novel signaling behavior could indicate the owls' current reproductive status to potential intruders, such as other territorial owls or non-breeding floaters. Faeces and prey feather markings may also advertise an owl's reproductive status or function in mate-mate communication.

**Conclusions/Significance:**

We speculate that faeces marks and plucking may represent an overlooked but widespread method for communicating current reproduction to conspecifics. Such marking behavior may be common in birds, and we may now be exploring other questions and mechanisms in territoriality.

## Introduction

Many animals mark focal elements of their home ranges with different kinds of materials. Spiders [1], salamanders [2], fish [3] and birds (e.g. [4]–[7]) use conspicuous visual and/or olfactory objects as a defense against predators, and to attract potential prey and mates. This territorial marking represents an extended form of display for some species. Moreover, many species of mammals demarcate their territory by faecal marks [8]–[10]. Faeces may represent an ideal substance for marking, because it has a minimal energetic cost to the signaler [11], and can continue to indicate possession of a territory when the owner is occupied in activities other than territorial defense. However, because faecal marking is constrained by faecal production, territorial individuals should prioritize the marking of positions that have the highest value as territorial signals [12].

Eagle owls (*Bubo bubo*) have been reported to use visual signaling in intraspecific communication, both in a territorial context (there is a white badge on the throat, which is repeatedly exposed at each call and is only visible during vocal displays), and in a parent-offspring context (a white border of feathers appears at the edges of eagle owls' mouths just before fledging, and becomes considerably less apparent upon dispersal) [13]–[15]. Consequently, we can surmise that this species is generally sensitive to visual communication, and could potentially employ various visual signals in other situations related to intraspecific interactions.

During the pre-laying period and throughout nestling period, large amounts of extremely visible white faeces and prey feathers appear on posts and plucking sites in the proximity of the nest site (a freshly scraped depression in the ground in which a female lays eggs). Although breeding owls use vocal displays to convey important territorial information, these signals have temporal limitations since they need to be produced actively and therefore require the owls' presence. Thus, it would seem useful to have additional, longer-lasting signals that would continue to indicate possession of a territory when owners are far from the nest. The nest represents the most crucial location within the owls' home range during the breeding season, as males and females frequently roost together close to the nest site for 2–3 months prior to egg-laying. Thereafter, the females remain in the nest for more than 2 months during incubation and through the first month of the nestling period, when the eggs and young chicks need continuous protection. During this period, eagle owls can show extremely aggressive behavior toward conspecifics (both neighbors and floaters searching for a breeding territory), with attacks sometimes proving fatal for owners and/or intruders (mainly the males) ([14], Penteriani and Delgado, personal observations).

Here, we test the novel hypothesis that faeces and feathers at plucking sites act as signal to territory intruders. The presence of highly visible signals like white faeces and bright feathers at plucking posts may indicate occupancy of territories. This could prevent floating individuals or neighbors from unintentionally approaching nest sites, thereby reducing the risk of potentially fatal aggressive encounters between competing males. Such territorial markings may have the additional advantage of indicating territory occupancy to potential intruders even when territory owners are far from the breeding site or are not involved in territorial displays (e.g. when they are hunting).

Here, we will investigate the following five hypotheses, although we must stress that several of our speculations are still working hypotheses that remain to be tested experimentally. If the faeces and prey remains in the areas surrounding eagle owl nests (Figure S1) are not used for signaling: (1) they should be randomly distributed within the home range and be conspicuous throughout the entire year, and (2) their presence on visible posts should be independent to the outcome of breeding (failure *vs*. success), as breeders stay close to the nests after breeding failures (Delgado and Penteriani, unpublished radiotracking data). Moreover, defecation posts and plucking sites should show similar patterns in the settlement areas used by non-territorial owls during dispersal. Such a comparison is possible because, after an early dispersal phase of actively searching for settlement areas, floaters inhabit stable settlement areas in which they show movement behaviors similar to those of breeders (Delgado, Penteriani and Nams submitted, Delgado and Penteriani in preparation). Furthermore, if there is no functional significance to marking with faeces and feathers, (3) one would expect faeces not to appear preferentially on dark substrates (increasing their conspicuousness) and to be in locations increasing their visibility. Because owls can expel faeces far from perches, one would expect faecal marks not to occur on rock faces with vertical walls (the contrary would indicate a functional significance to faeces visibility; Figure S2). If marking is random and environmental factors partially or completely remove faeces from a post, (4) one would expect them not to be replaced by fresh markings. As for prey remains, (5) they should be largely composed of the owl's main prey (the rabbit *Oryctolagus cuniculus*, which represents 68–86% of the diet by biomass in the study area; Delgado and Penteriani, unpublished results), even though the color of the rabbit fur represents a poor visual signal. Furthermore, the prey remains should be consistently located within or close to the hunting areas, where owls catch and eat most of their prey (Delgado and Penteriani, unpublished radiotracking results).

## Results

### Features of faecal marks and plucking sites during the year

Within the 20 studied home ranges, we located 194 defecation posts and 41 plucking sites. Posts with faeces and plucking sites were located 163.5±24.9 m (range = 0.5–603.7 m) and 178±56.3 m (range = 4.8–695.1 m) from the nest, respectively. Such posts were found within 0.5±0.7% only of the 50% core area (Figure S3) and started to appear in late September, during the renewal of territorial displays, increased until March, and decreased thereafter during the fledgling period (Figure S4). Because defecation posts and plucking sites are not refreshed after fledging (Figure S4), it seems unlikely that a large amount of faeces and feathers simply built up at these locations because the owls, for a variety of reasons, are selected resting points near the nest on which faeces and feathers accumulate by chance (i.e. they are not using excrements or feathers as signaling items). But supplementary experimental evidences are needed to completely discard alternative hypotheses. However, it is important to note that we never observed post refreshing after fledgling or during the post-fledging dependence period (Campioni, Delgado and Penteriani, unpublished radiotracking results on 27 breeders), when the following holds true: (a) both parents do not show important modifications of their space use and continue to stay relatively close to the nest; (b) small differences in the space use due to young displacements are not followed by the appearance of new marked posts; (c) diurnal roosts continue to be generally located in the area surrounding the nest (i.e. territory owners start and end their activity close to the previously marked posts); and (d) such posts are still located within the 50% core area of owl movements.

The plucking sites of radiotagged owls were located at a mean distance of 178±56.3 m from the nest. Only 10 out of 91 (10.9%) recorded hunting events occurred within the plots calculated on the basis of the mean distance of the plucking sites to the nests; the others were at a mean distance of 1235.7±1350.4 m. In other words, the eagle owls generally hunted and ate their prey at a distance from the nest, but all plucking sites were concentrated close to the nest (t = 10.12, df = 131, P = 0.003). Similar to the locations of the defecation posts, the plucking sites were located at the highest, most visible points of the valley slopes (χ^2^ = 21.00, df = 2, P = 0.0001). After incubation, the owls ceased plucking on conspicuous posts and more frequently plucked their prey on the ground.

### Relationship between appearance of defecation posts/plucking sites and owl breeding status

Immediately after a breeding failure (n = 33), the conspicuous plucking sites disappeared and the marked posts were not renewed, even though the parents continued to move and roost close to the nest. The plucked preys or faeces that were observed after breeding failure were generally on the ground, and not in prominent locations.

Finally, any faeces-marked rocks or prominent plucking sites were never found in the settlement areas frequented during dispersal by 33 tagged juveniles. Non-territorial individuals do not leave their faeces or prey remains on visible posts, even though they frequently used the same posts within their settlement area home ranges (Delgado, Penteriani and Nams submitted).

### Conspicuousness of the faeces with regard to the substrate, their position, and the posture of defecating owls

Compared to bright substrates (n = 22), an unambiguous prevalence (χ^2^ = 102.76, df = 1, P = 0.0001) of faeces on dark substrates (n = 158) was recorded, even though bright rocks prevailed in the examined breeding sites (χ^2^ = 122.66, df = 1, P = 0.0001; Figure S5).

Defecation posts were largely located on the highest points of valley slopes (χ^2^ = 120.70, df = 2, P = 0.0001) and on positions that made them easily detectable from neighboring territories or by non-territorial individuals moving across the main valley (χ^2^ = 161.40, df = 2, P = 0.0001; Figure S6). Although it was not possible to collect quantitative data on the signaling effort of each territory owner, eagle owls with nearby neighbors and/or in situations of very high breeding territory density seemed to mark more posts compared to owners of territories located relatively far from their nearest neighbors.

Due to the distance at which eagle owls generally squirt their faeces (20.1±10.8 cm; n = 108), isolated defecation rocks with abrupt and vertical shapes (the most common type of marked posts, see Figure S2; χ^2^ = 82.67, df = 1, P = 0.0001) should not show faecal marks if marking was due by coincidence. However, we did not test for the possibility that owls may defecate in a different manner in high visibility sites versus less visible sites.

### Refreshing of defecation posts

Owls responded rapidly to changes of faecal marks on defecation posts (Figure S7). In fact, the mean time elapsed between the experimental covering of the faeces and the appearance of new faecal marks on the same post was of 2±1.8 days. In 18 (30%) of defecation posts, new faeces appeared in <24 hours. Faeces never reappeared on the random posts.

### Relationship between the owls' main prey and prey on plucking sites

We never found rabbit remains on plucking sites, whereas many rabbit remains were observed on the ground, in low-visibility positions. All prey species found on plucking sites were birds endowed with highly visible (white or bright) feathers (Figure S8); these included the azure-winged magpie (Cyanopica cyanus, n = 3), barn owl (Tyto alba = 2), Larus spp. (n = 1), little egret (Egretta garzetta, n = 3), red-legged partridge (Alectoris rufa, n = 14), short-eared owl (Asio flammeus, n = 1) and wood pigeon (Columba palumbus, n = 17). The low frequencies of the species recorded on the plucking sites in the owl diet (mean range = 0.8–6.9%) clearly show that they are among the less common prey in the study area.

## Discussion

In an unpredictable natural world in which some birds are capable of masticating vegetables to paint a saliva-plant mixture on their bowers [7], or arrange feathers to decorate their nests in a non-random way with respect to their reflectance [6], we present preliminary evidence suggesting that owls may use faeces and prey feathers to signal their breeding status to conspecifics. In contrast to breeders, subordinate, non-territorial floaters never mark their territories, even though they often reside in the same settlement area for several months. As we would expect for a signaling behavior that has evolved to maximize signal strength relative to the background environment [16], the data in this study suggest that eagle owls preferentially leave white faeces on the darkest and most detectable surfaces, and preferentially leave prey species with conspicuous plumage at highly visible plucking sites.

Granted, these signs might also contain useful information for predators. However, eagle owls can likely afford to give away the location of their nest sites in such a prominent manner because adult eagle owls almost have no natural predators, and females are present in the nest with their offspring throughout most of the nestling period.

Marking with white faeces and prey remains shows many similarities with mammal scent marking [17]. Some of the faecal marks are only visible from the nests (Figure S9), suggesting that they could also signal the owl's reproductive state or function in mate-mate communication (e.g. choice of the nest placement), in a manner similar to that seen for scent marks in a number of mammalian species [17], [18]. In such a context, we can not exclude the possibility that the faecal markings could provide a signaling function similar to that conveyed by the transport of green material to the nest (especially for owls that do not carry nesting materials), which in some bird species serves as an intersexual signal for nest occupation [19]–[21]. In mammals [17], [22], the location of marked defecation posts allows them to be detected at some distance, alerting animals to the presence and location of an occupied territory. Moreover, the owls' defecation posts and plucking sites are situated relatively close to each other, potentially maximizing their chance of detection by intruding individuals [17], [22]. These markings could therefore function as an effective deterrent to neighboring owls or floaters. However, this hypothesis would need to be confirmed using field experiments. Scent marking, defecation posts and plucking sites enable the signaler to leave messages that are long lasting and can be read later by conspecifics [23], suggesting that they could have evolved as broadcast signals used for network communication. Finally, similar to transient mammals with no mate or territory [24], eagle owl floaters do not mark their settlement areas with faeces or prey remains.

Faecal marks and plucking sites only occur in close proximity to the nest, which is the most actively defended area [14]. The Ownership Hypothesis for mammalian scent marking [25] predicts that some markers are designed to claim ownership or exclusive use of focal resources or sites. Under this hypothesis, and as observed in the present study in eagle owls, markers are not necessarily expected to occur on the boundaries of the home range, because some portions of the home range may overlap with neighboring territories ([26] and unpublished radiotracking data). The shift from a peripheral to a central position of marks within the home range has been attributed to the size of the territory/home range or the energetic and time constraints limiting border patrolling behavior [27], [28]. However, although some of these factors may be true for eagle owls, the concentration of faecal marks only around active nests may indicate a stronger territoriality in this area (home range overlap never occurs in the 50% core area, [26]). This occurs in eagle owl territories despite the relatively small territory and home range sizes (577±522 ha, n = 9 owls), which should favor peripheral home range boundary marking.

The effective transmission of a signal requires the signaler to assess and react to changes in the signal [29]. We found that eagle owls rapidly detected a change in faecal marks and compensated by re-marking the same location with fresh faeces, suggesting that owls are capable of controlling their displays through behavioral compensation.

Similar to the white patches of eagle owl plumage reported previously, we hypothesize that the faecal marks and plucking sites described herein may act as previously unrecognized visual signals. Because both white faecal marks and plucking spots are well-known features of both diurnal and nocturnal bird of prey territories [19], it is tempting to speculate that such signaling behavior may represent an overlooked but widespread manner in which such species (and perhaps others) communicate territorial ownership to conspecifics (Figure S10).

The data presented herein provide a baseline for further testing of this hypothesis. However, to obtain stronger evidence on the intriguing idea that eagle owls use faeces and prey feathers to signal current reproduction (i.e. excluding that defecation marks may have a value as information without being an evolved signal), we will need to perform further experimental studies and behavioral observations that: (a) examine whether faeces and feathers provoke specific behavioral reactions and what functional significance these behavioral reactions have; and (b) allow us to completely discard the possibility that some of the observed patterns could simply reflect increased vigilance by parents on preferred resting spots near the nest (e.g. vantage points from which parents prefer to keep watch).

To conclude, we consider important to highlight the speculative nature of our paper. In our opinion, its main importance is to present the occurrence of some intriguing patterns of animal behavior that may open many interesting questions that can/should be addressed in future research.

## Materials and Methods

### About the study species

The eagle owl is the largest owl in the world; it has a body mass between 1.5 (males) and 4 kg (females). There are no differences in plumage characteristics between the sexes. This monogamous, long-lived species is highly territorial throughout the year, with the males performing most of the territorial defense [30], [31]. Eagle owl vocal behavior is associated with intra- and intersexual territorial disputes, as well as with courtship behavior [14]. The species can reach very high densities (in our study population there were 35 breeding pairs per 100 km^2^) and engage in complex spatio-temporal individual interactions. The species is a generalist predator occurring in a wide range of habitats throughout the Palearctic regions.

### General methods and radiotracking procedures

To test the hypothesis that owls use visual sign posts to mark territory occupation or focal placements within their home range, we recorded some essential characteristics of both faecal marks and plucking sites in 20 breeding territories of eagle owls (2004–2005) and in the settlement areas frequented by 33 floaters (2003–2005) in the Sierra Norte of Seville (south–western Spain). All defecation posts and plucking sites were located using GPS. We used the GIS ArcView 3.2 software to analyze the spatial characteristics of marked posts. Both defecation posts and plucking sites were plotted on 1∶10000 maps. Additionally, during the 2000–2005 breeding seasons, we looked for possible relationships among the temporal and spatial patterns of faeces and prey remains, and owl breeding performance. For some analyses, we used a sub-sample of both the 20 breeding territories and the total amount of defecation posts and plucking sites. To balance the sample size, we used the same number of defecation posts or plucking sites for each breeding pair.

To test some hypotheses, we required information from radiotagged owls. Marked individuals were equipped with 30-g harness-mounted backpacks, with a mercury posture sensor that allowed us to record the rhythms of activity during the night. The weight of the tags corresponded to less than 3% of the weight of the smallest adult male (1550 g) in our eagle owl population (1667±104.8 g, n = 9 adult males). Capture was made by simulating a territorial intrusion with a combination of taxidermic decoys and a net (see [14]). The capture and handling of breeding owls was always very safe; the owls were immediately removed from the net upon capture, and stayed motionless when handled. We trapped and marked owls under Junta de Andalucía–Consejería de Medio Ambiente permits No. SCFFS-AFR/GGG RS-260/02 and SCFFS-AFR/CMM RS-1904/02. In 4 years of continuous radio tracking of more than 50 eagle owls (both breeders and non-breeding juveniles), we never recorded a possible adverse effect of a backpack on an individual or its breeding performance (Delgado and Penteriani, unpublished data). The backpacks were not removed after the study because it is difficult to re-trap individuals (Penteriani and Delgado unpublished data). Owls were tracked individually in continuous radiotracking sessions, during which we recorded all individual movements from one hour before sunset to one hour after sunrise. Instead of sampling at fixed time intervals, we located an individual each time it moved. This strategy was chosen because: (1) it is more biologically meaningful to record the real-time rhythms of activity; and (2) this avoided problems of over- or undersampling due to the inter-sampling time interval, consequently guarding against a possible lack of independence of successive observations.

### Testing the hypotheses

#### Prediction 1: spatial and temporal distribution of faecal marks and plucking sites within the home range

Throughout the entire year, we surveyed the nest areas in 20 breeding territories, and recorded the spatial and temporal distribution of faecal marks and plucking sites. Our study area represents an exceptional site for testing this type of visual signaling in owls because there are no other large bird of prey species in the area, thereby limiting confusion between eagle owl faecal sites and those of other species. We characterized home ranges by identifying 50%, 70% and 90% core areas (Adaptative Kernel Contouring method) using the “Home Range” extension of the ArcView 3.2 software package. This information was used to compare the area delimited by the posts with faeces and prey remains with the 50% core area.

We performed continuous radiotracking three times a week throughout the entire year, for approximately 350 nights (>3500 hrs) of continuous radiotracking of more than 50 radiotagged owls between 2003–2006. We identified hunting behaviors from among other activities (e.g. plumage preening of a roosted individual) when: (1) the tag pulse increased its frequency and its volume changed due to the variation of the owl-antenna distance, signaling that an individual was flying to the hunting area after roosting for a long time (i.e. ambushing prey); and (2) the frequency of the pulse increased and decreased rhythmically, the volume remained unchanged (indicating that the individual was stationary), and the individual was not calling (vocal displays generated similar patterns in the frequency pulses), signaling that the owl had successfully hunted and was eating the prey. After locating hunting areas, we calculated: (a) the distance covered between the nest and the mean point of each of these areas, which was compared with the distances between the nest and the plucking sites; and (b) the frequencies of hunting events within a radius equal to the mean distance between the nest and the plucking sites. Finally, two parameters were chosen to describe the visibility of plucking sites: (1) their height relative to the valley slope (three classes, obtained by dividing the slope into thirds: the bottom, middle and upper parts of the slope); and (2) their position with regard to the: (a) main valley, (b) neighboring territories of conspecifics and (c) main valley and neighboring owl territories. χ^2^-tests were used to compare frequencies between categories.

#### Prediction 2: relationship between appearance of defecation posts/plucking sites and owl breeding status

From 2000 to 2004, we checked 106 breeding attempts in the study area. During each breeding season, we visited the home range before, during and after egg incubation, and recorded the presence/absence of faecal marks and plucking sites.

From 2003 to 2005, we radiotagged 33 owlets from 11 nest sites (2003: n = 6; 2004: n = 10; 2005: n = 17) when they were approximately 35 days old. Because the young were still growing at this time, the backpacks were adjusted so the Teflon ribbon could expand, allowing for increases in body size. The capture of owlets was always very easy and safe, because owls of this age stay motionless when humans approach. Starting from the beginning of dispersal, and during the entire year, we surveyed the temporary settlement areas used by tagged floaters (i.e. individuals that were already independent from their parents and were searching for territories), and noted the presence/absence of defecation posts and plucking sites.

#### Prediction 3: conspicuousness of faeces with regard to the substrate, their position, and the posture of defecating owls

We randomly selected a sub-sample of 180 rocks marked with white faeces (n = 9 rocks per n = 20 pairs), and classified them as dark (e.g. brown and dark orange) and bright (e.g. white-grayish) substrates. The random sample was obtained by progressively numbering each faecal post and randomly extracting nine of them from each breeding territory. To control for the general color of substrates in the breeding area, we determined a control site for each defecation post on the basis of the predominant color of the rocks surrounding the nesting site. We excluded a potential sampling bias due to the fact that excrement on dark rocks is more conspicuous. In fact, as clearly shown in Figures S1, S2, S6, S7 and S9, faecal marks are also evident on bright rocks, although there may be a preference for marking the darkest portions of lighter rocks (see Figure S5). The occurrence and frequency of faeces on the defecation vs. control substrates were compared using χ^2^-tests.

We measured and described the visibility of the defecation posts by calculating the same two parameters used for plucking sites (Prediction 1). χ^2^-tests were used to compare frequencies between these classes.

We recorded the shape of the defecation posts, grouping them into two categories: rectangular rocks with abrupt, vertical walls; and rounded or triangular rocks with less angular wall slopes. Eagle owls squirt their faeces almost horizontally for some distance, meaning that the marks tend to land relatively far from the perch sites. Thus, vertical rock faces are less likely to receive faecal marks (the typical eagle owl post, as shown in all of the SI Figures, demonstrates this). It could therefore be argued that the vertical faces that receive faecal markings are being used for the purpose of territorial marking. To explore this possibility, we also measured the distance at which eagle owls are able to expel their faeces, by calculating the distance between an owl perch and the nearest margin of faecal stripes, using 5 eagle owls in a raptor rescue centre.

#### Prediction 4: refreshing of faecal marks

In each of the 20 breeding territories, we removed the faeces from three randomly selected posts (n = 60) and three random locations (n = 60; October–December 2004, before egg-laying). The random locations were places where faeces were found on the ground, or on a post covered by vegetation, thereby making the faeces low-visibility and unlikely to be used for marking. In the morning, we covered the faeces at both locations by spray-painting the marks with a paint color similar to the background color. Each location was visited daily for 15 days post-treatment to check for fresh faecal markings.

#### Prediction 5: relationship between main owl prey and prey on plucking sites

We recorded the prey species on the plucking sites to check if the species distribution on the posts reflected that in the owls' diets (as previously analyzed in the study area). During the study period, all territories were sampled in all years to avoid a potential bias associated with possible temporal variations in diet [32]. We determined diet by repeated nest visits to collect prey remains and pellets and by direct observations at sunset and sunrise. The combination of different methods to determine diet may yield more accurate estimates of the overall diet compared to the use of just one method. Prey remains and pellets were identified by macroscopic comparison with reference collections. We pooled pellets from individual visits into a single sample for analysis. The presence of different prey types in the samples was recorded, but no attempt was made to quantify the number of individuals. To avoid duplication of prey (i.e. in remains and pellets), items found in pellets were used only if they had not been found as remains during the same visit.

## Supporting Information

Figure S1 Pictures showing details of eagle owl faecal markings.
(1.30 MB PDF)

Figure S2 To increase the conspicuousness of faecal signaling, owls need to mark the most prominent rock surfaces.
(0.28 MB PDF)

Figure S3 An example of the spatial distribution of faecal markings within an eagle owl's home range.
(0.07 MB PDF)

Figure S4 Temporal pattern of the appearance of defecation and plucking sites, which generally become visible during the pre-laying season, and remain visible up until the fledgling period.
(0.31 MB PDF)

Figure S5 Some examples of preferential use of darkest substrates for eagle owl faecal marking.
(0.35 MB PDF)

Figure S6 Both faecal marks and plucking sites are located in positions with increased conspicuousness, such as dominant places and the highest points of valley slopes. Some marks also appear at the entrance of the valley in which the nest is located.
(5.80 MB PDF)

Figure S7 Examples of faecal marks being refreshed after we experimentally obscured them with spray paint. Generally, the eagle owls returned to re-mark within one to two nights of the experimental covering. In several cases, faeces were scattered at exactly the same position that had been previously marked.
(0.89 MB PDF)

Figure S8 Pictures showing the significant contrast between the bright feathers and the dark surface of the plucking site. All the prey species on plucking sites were birds with highly visible feathers.
(0.59 MB PDF)

Figure S9 Some faecal marks were only visible from the nest, not the surroundings. In such a context, they could act to signal reproductive status between the male and female of the breeding pair.
(0.24 MB PDF)

Figure S10 In the absence of dominant posts, eagle owls use different locations to signal their breeding status, such as trunks, fences, poles and human structures. Faecal marks and plucking sites could also function as visual signals in other avian species, such as the Little Owl, Athene noctua.
(0.29 MB PDF)
