# Supplementary material for: Owls May Use Faeces and Prey Feathers to Signal Current Reproduction
Source: PLoS One. 2008 Aug 20;3(8):e3014. doi: 10.1371/journal.pone.0003014 (PMC2507733; doi:10.1371/journal.pone.0003014)
Supplement: Figure S1 — Pictures showing details of eagle owl faecal markings. (1.30 MB PDF) [file pone.0003014.s001.pdf]

S1: PATTERNS OF DEFECATION SITES AND FAECAL MARKS

Cliff faces showing eagle owl faecal markings (A - K)

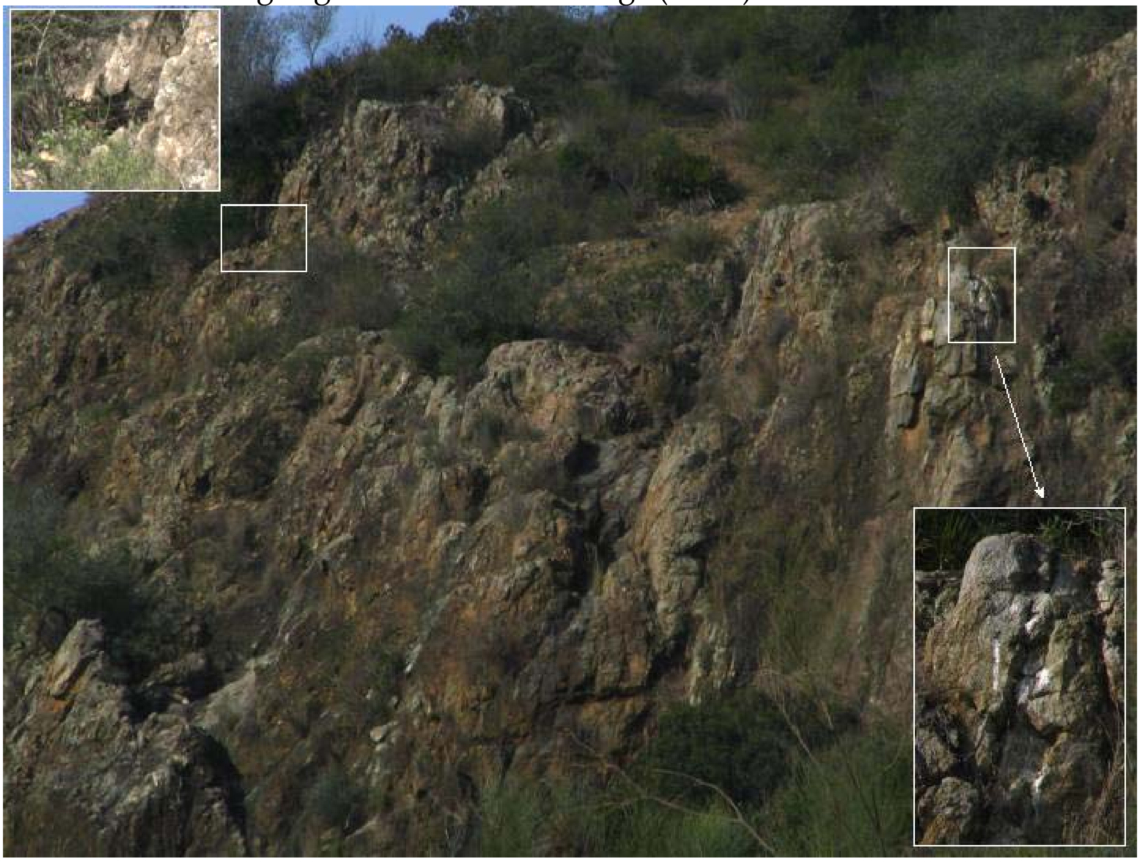

A

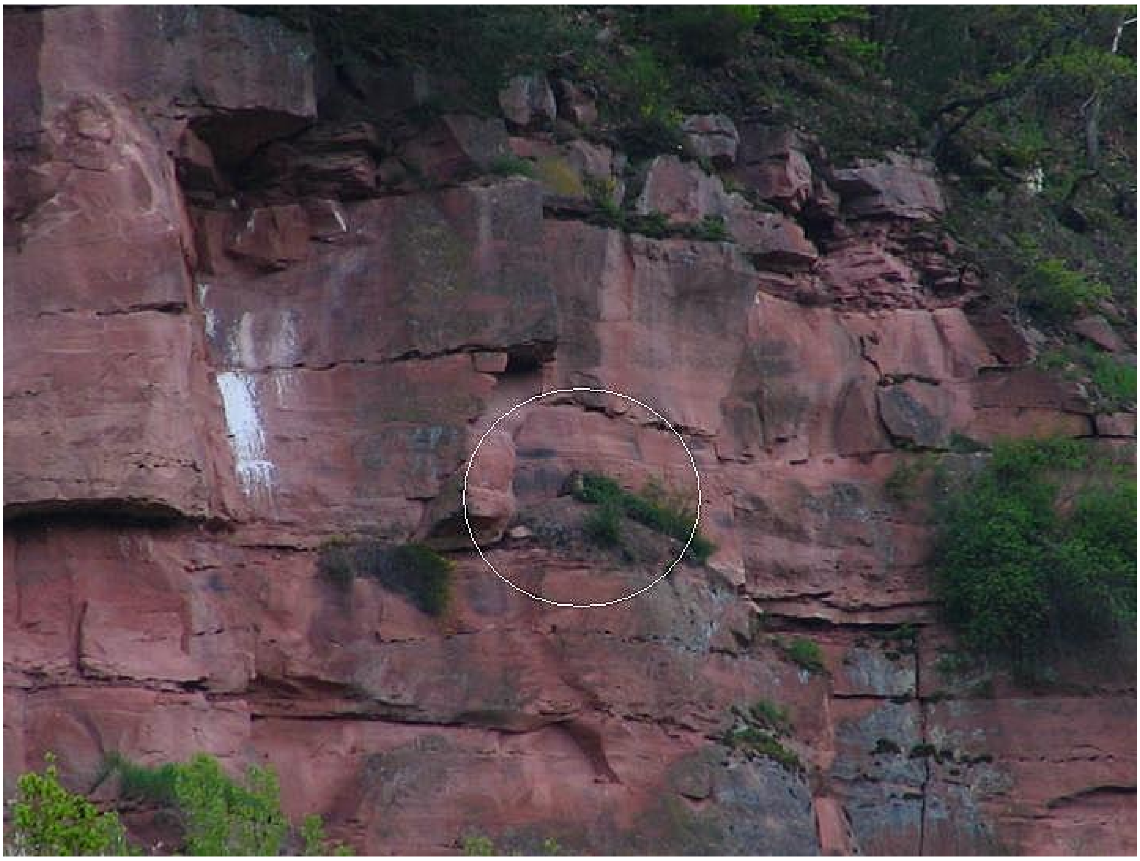

B

S1: PATTERNS OF DEFECATION SITES AND FAECAL MARKS

Defecations sites are always close to active nest sites, as illustrated by the white markings in photo A, which provides details of both the incubating female and the faecal marks, and in B, in which the highest amount of white marks are seen to the left of the female resting in the nest.

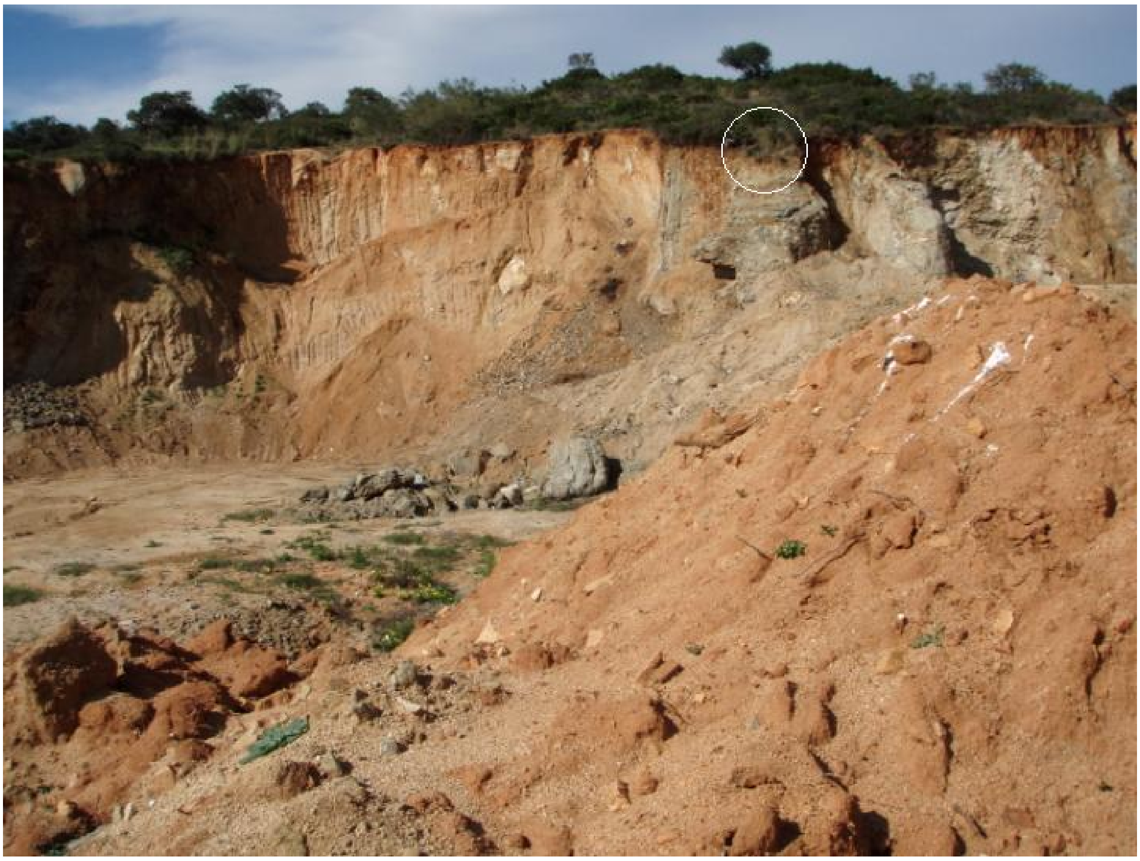

C

Faecal marks are often found just in front of the nest chosen for breeding (circles in C, D and E), and in many cases the nest depression is visible from the defecation site (circle in D). Owls show a preference for marking huge vertical surfaces. The vertical nature of defecation sites supports our hypothesis of faecal signaling, because normal defecation would not cause the “painting” of such types of rocks (see also text and SUPPORTING FILE 2).

S1: PATTERNS OF DEFECATION SITES AND FAECAL MARKS

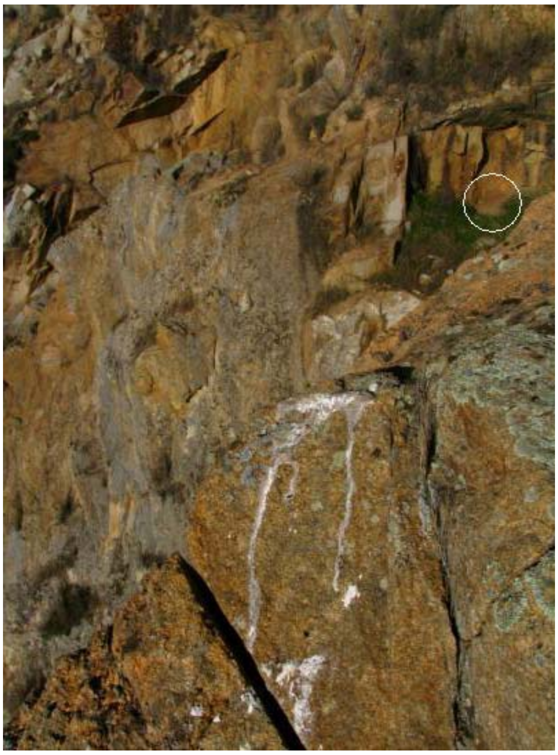

D

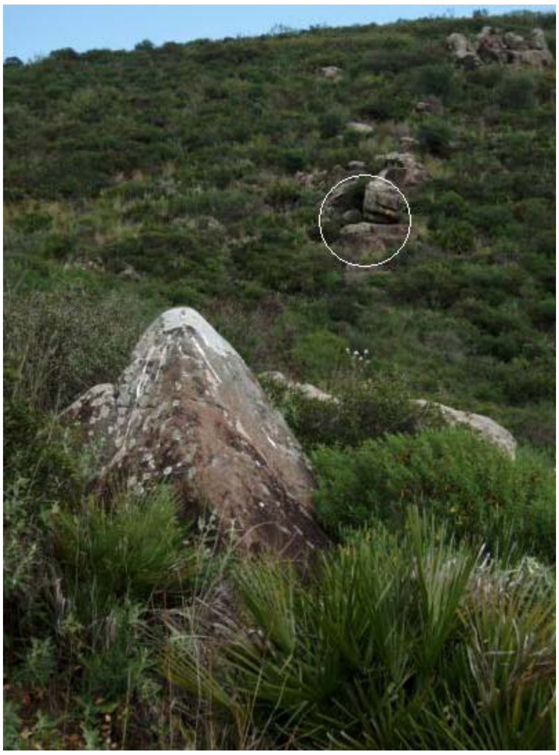

E

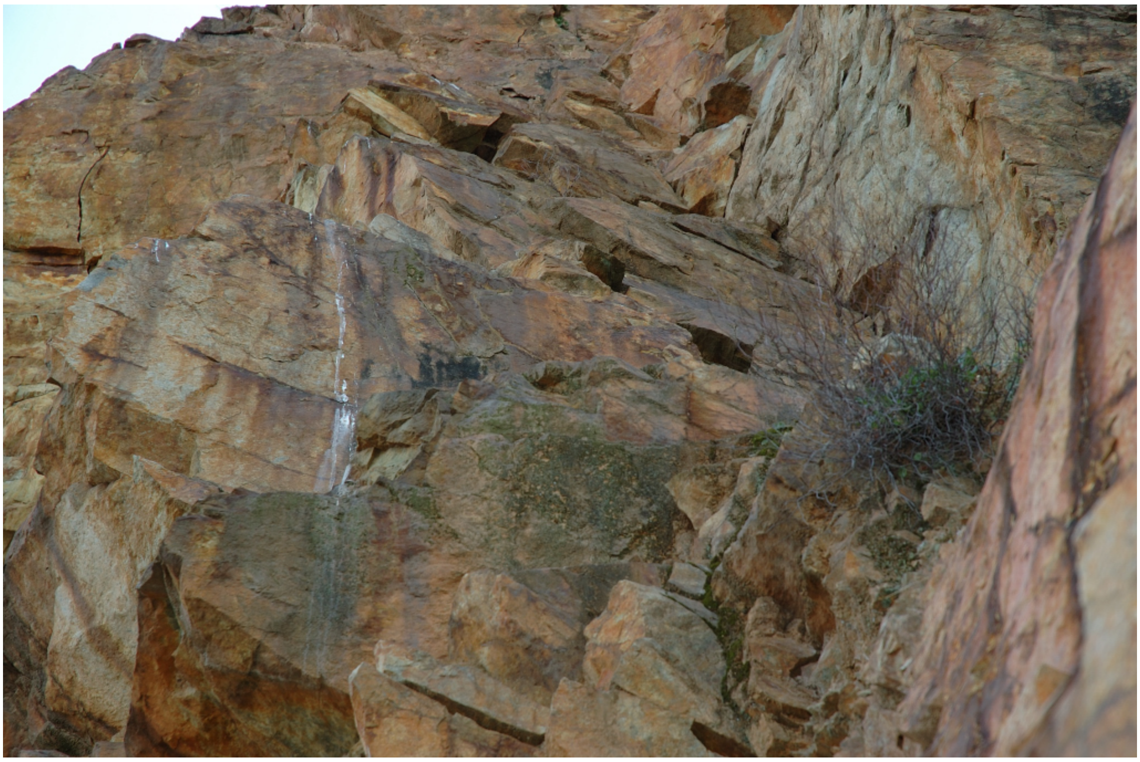

F

S1: PATTERNS OF DEFECATION SITES AND FAECAL MARKS

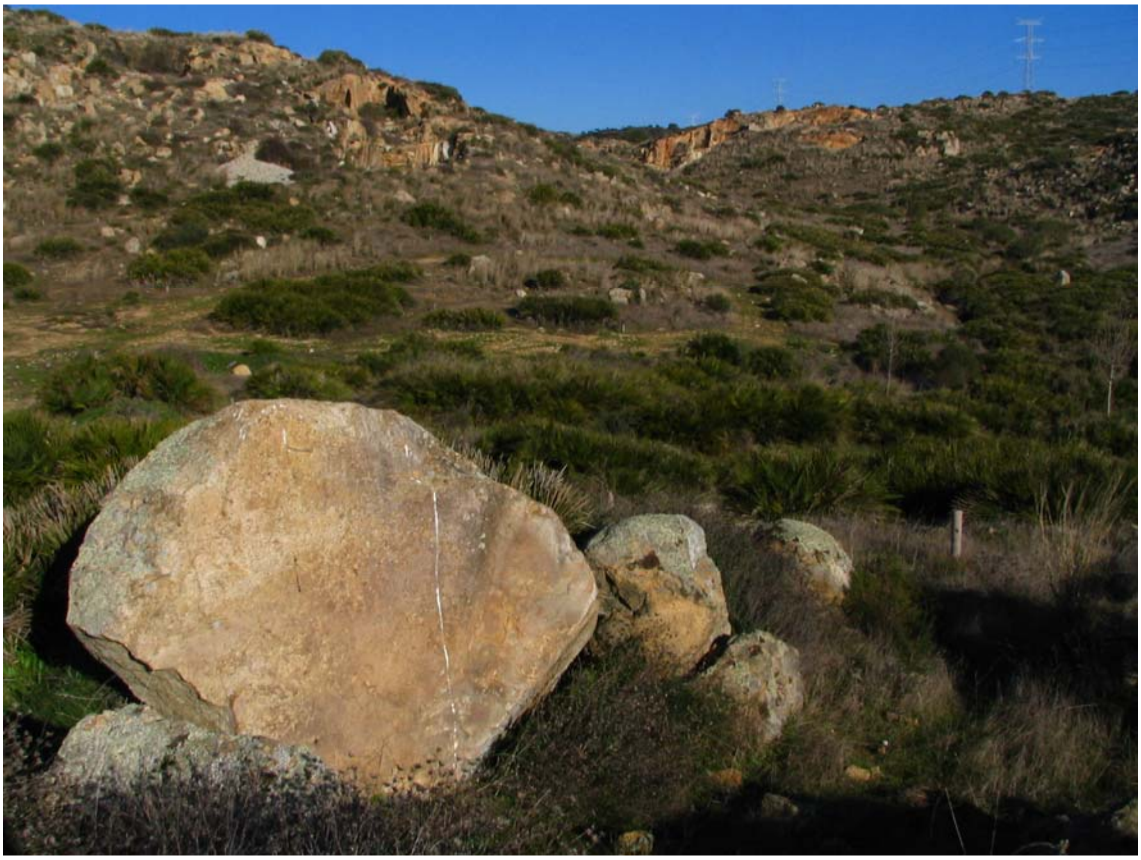

G

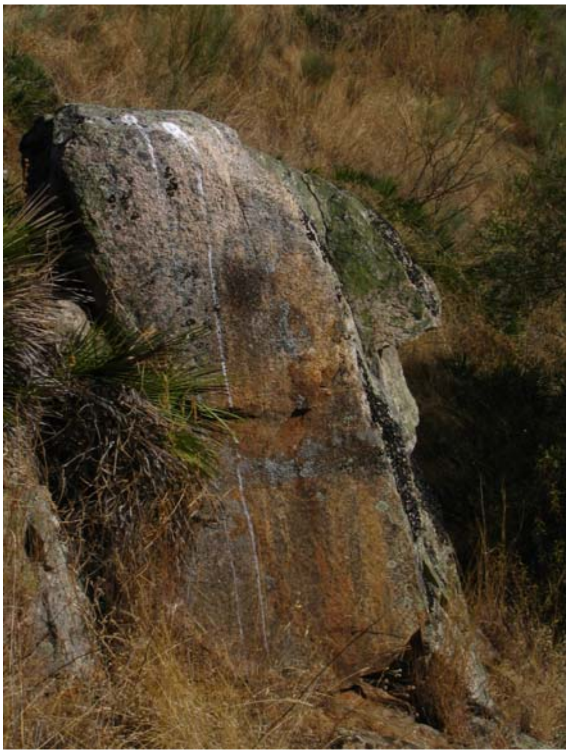

H

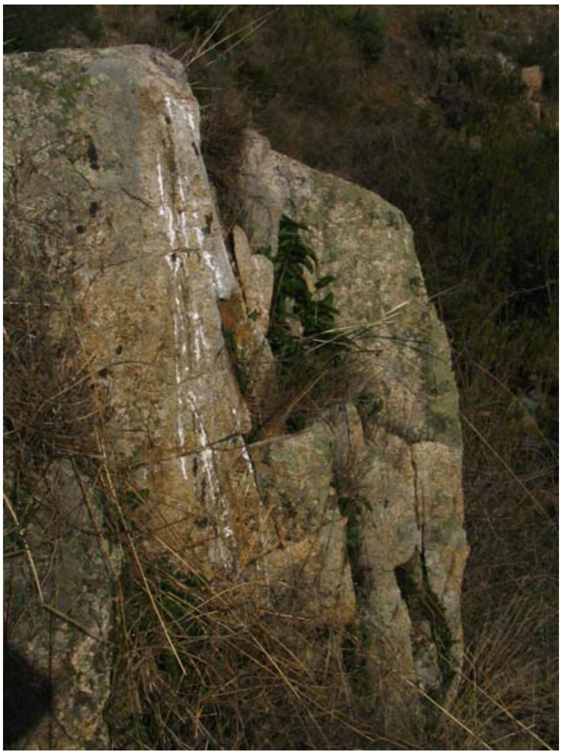

I

S1: PATTERNS OF DEFECATION SITES AND FAECAL MARKS

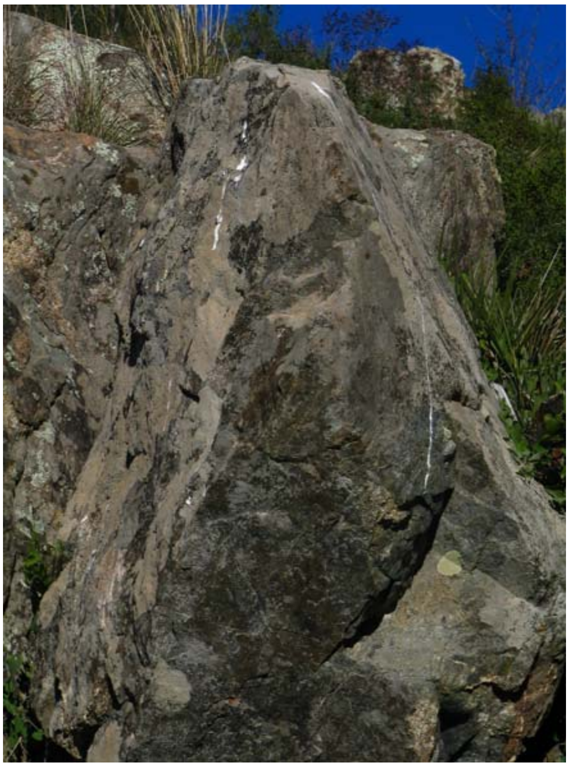
J

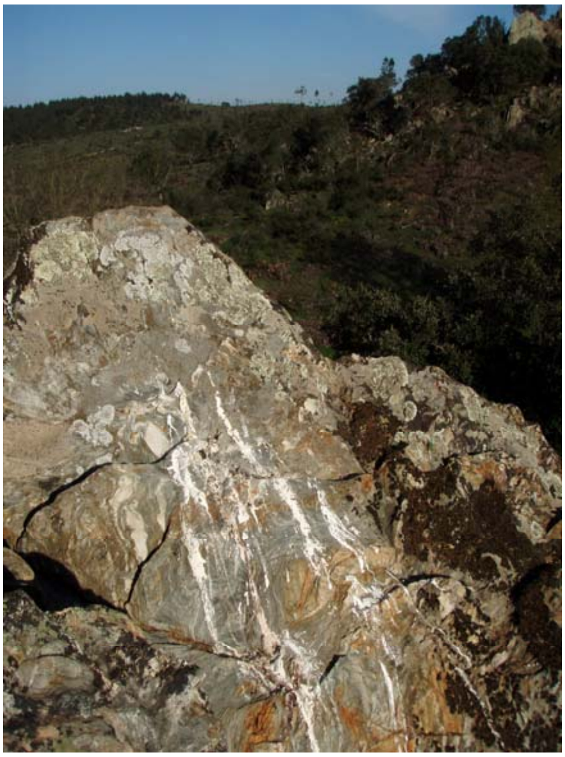
K
