# Supplementary material for: Owls May Use Faeces and Prey Feathers to Signal Current Reproduction
Source: PLoS One. 2008 Aug 20;3(8):e3014. doi: 10.1371/journal.pone.0003014 (PMC2507733; doi:10.1371/journal.pone.0003014)
Supplement: Figure S2 — To increase the conspicuousness of faecal signaling, owls need to mark the most prominent rock surfaces. (0.28 MB PDF) [file pone.0003014.s002.pdf]

S2: OWL POSITIONS ON FAECAL POSTS

To give the faeces greater visibility (e.g. when distinguishing the main breeding territories of neighboring pairs), owls need to mark the most prominent surfaces of the target rocks. This causes individuals to perch in a way that is not usual when they roost, i.e. without clear views of their surroundings (as for example in B and C). This owl posture clearly shows that it is marking its territory with faeces.

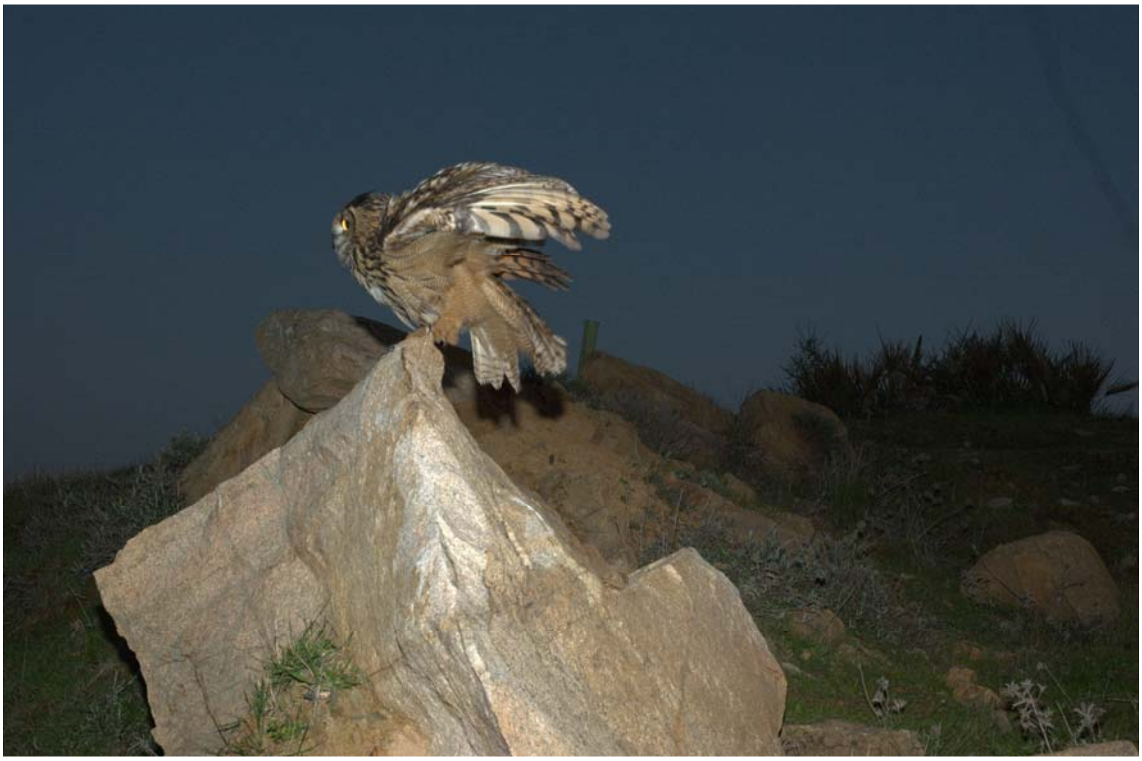

A

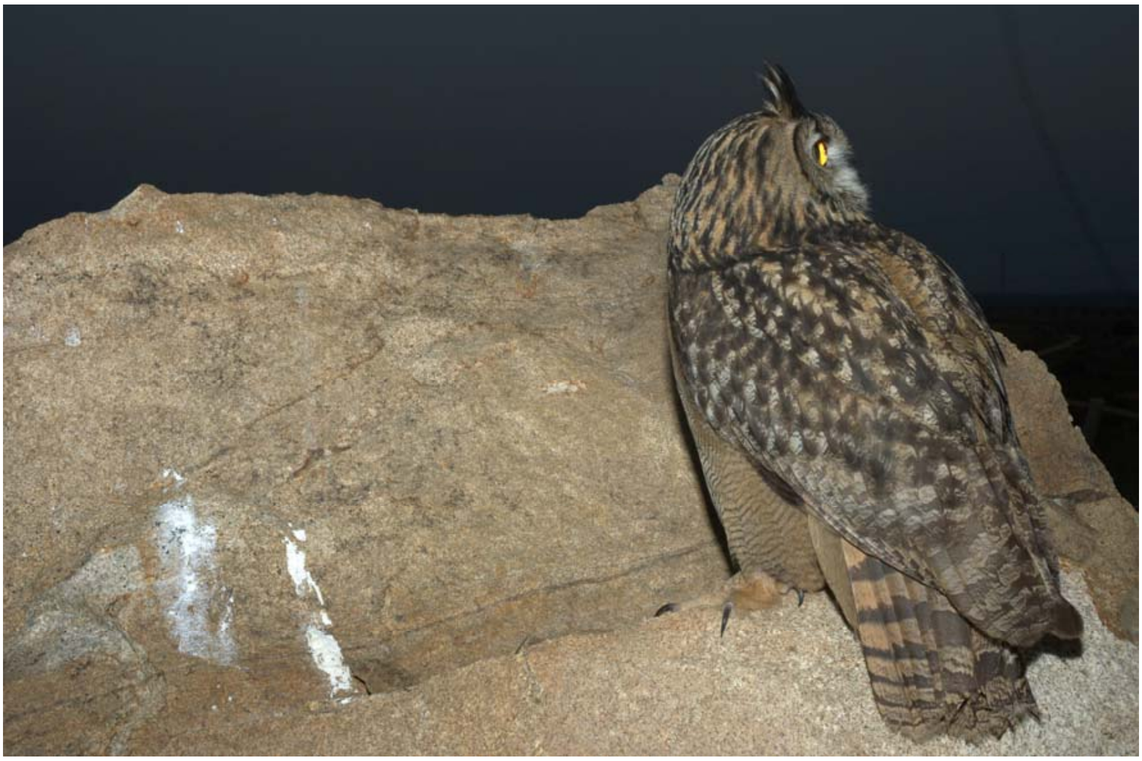

B

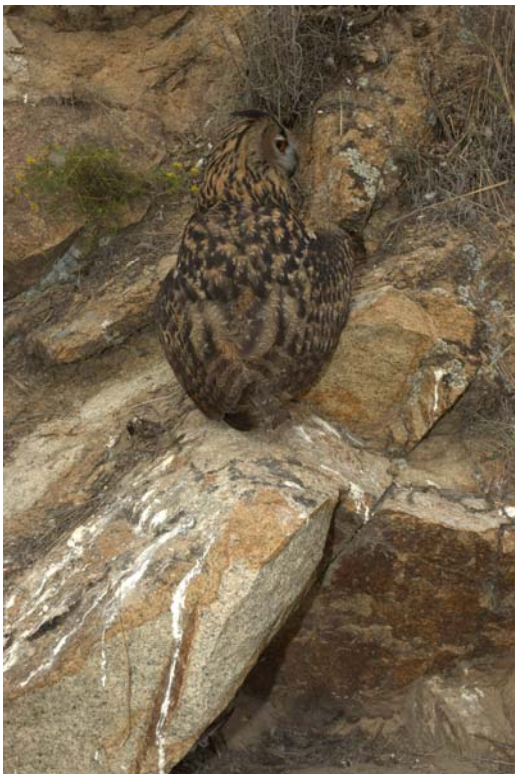

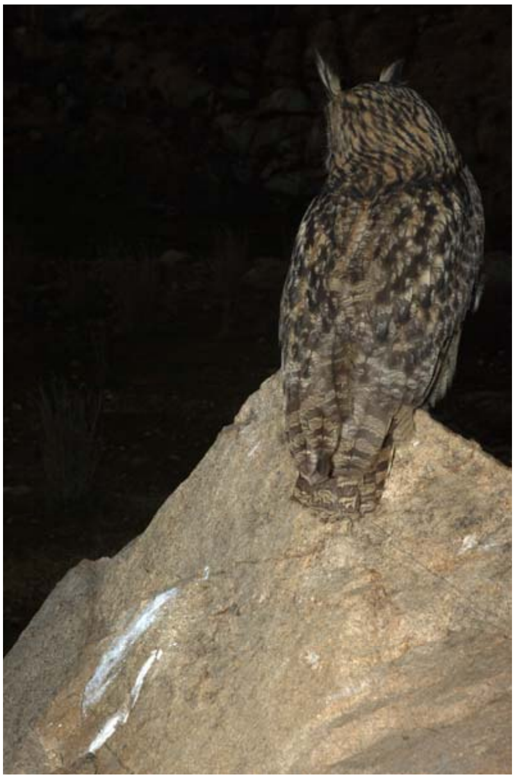

C **D**
