# Supplementary material for: Owls May Use Faeces and Prey Feathers to Signal Current Reproduction
Source: PLoS One. 2008 Aug 20;3(8):e3014. doi: 10.1371/journal.pone.0003014 (PMC2507733; doi:10.1371/journal.pone.0003014)
Supplement: Figure S3 — An example of the spatial distribution of faecal markings within an eagle owl's home range. (0.07 MB PDF) [file pone.0003014.s003.pdf]

S3: SPATIAL DISTRIBUTION OF FAECAL MARKINGS

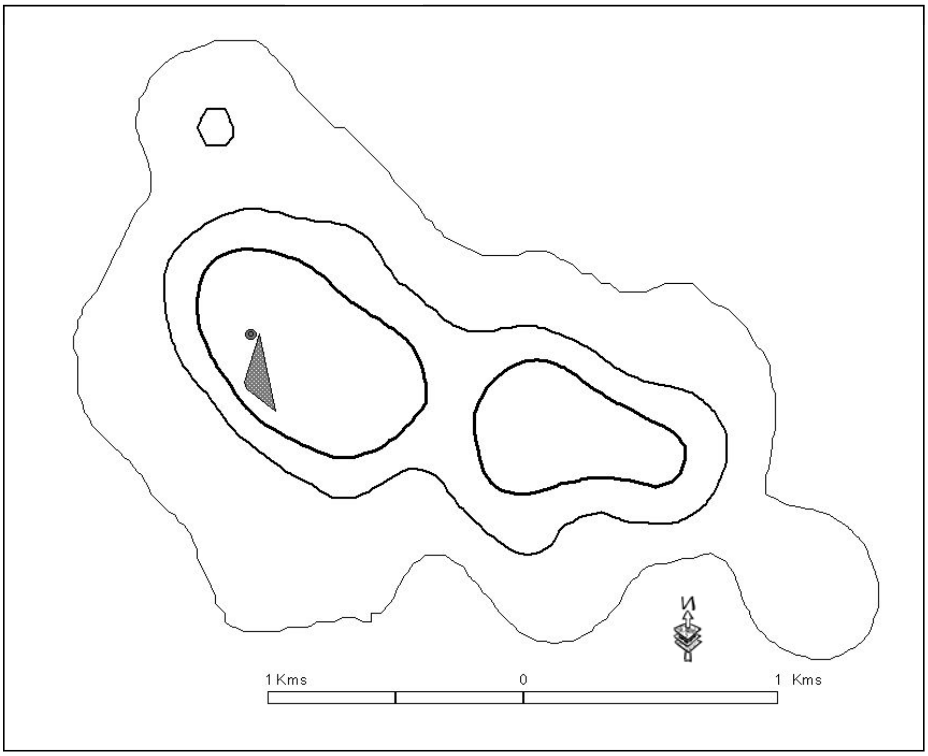

An example of the spatial distribution of faecal markings (grey triangle) within the home range of a breeding eagle owl. Defecation sites only occur in a very limited portion of the core area of the whole home range, namely the region closely surrounding the active nest (grey circle).
