# Supplementary material for: Owls May Use Faeces and Prey Feathers to Signal Current Reproduction
Source: PLoS One. 2008 Aug 20;3(8):e3014. doi: 10.1371/journal.pone.0003014 (PMC2507733; doi:10.1371/journal.pone.0003014)
Supplement: Figure S4 — Temporal pattern of the appearance of defecation and plucking sites, which generally become visible during the pre-laying season, and remain visible up until the fledgling period. (0.31 MB PDF) [file pone.0003014.s004.pdf]

S4: TEMPORAL PATTERN OF THE APPEARANCE OF DEFECATION SITES

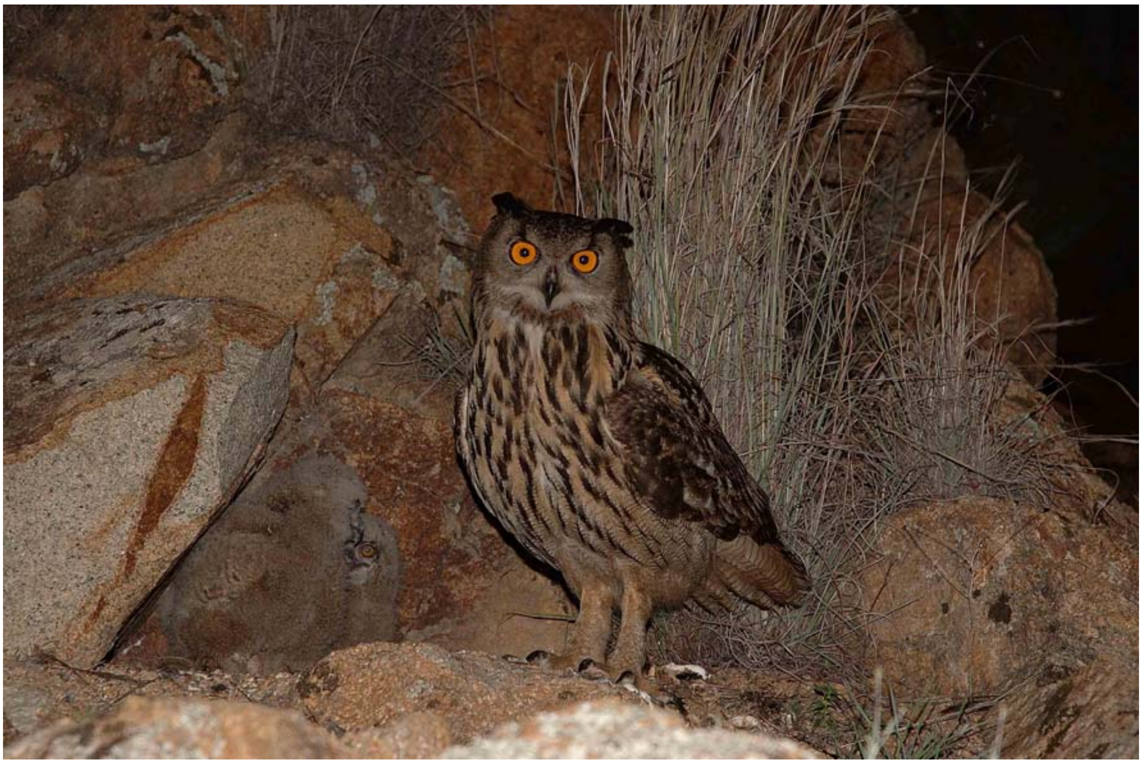

A

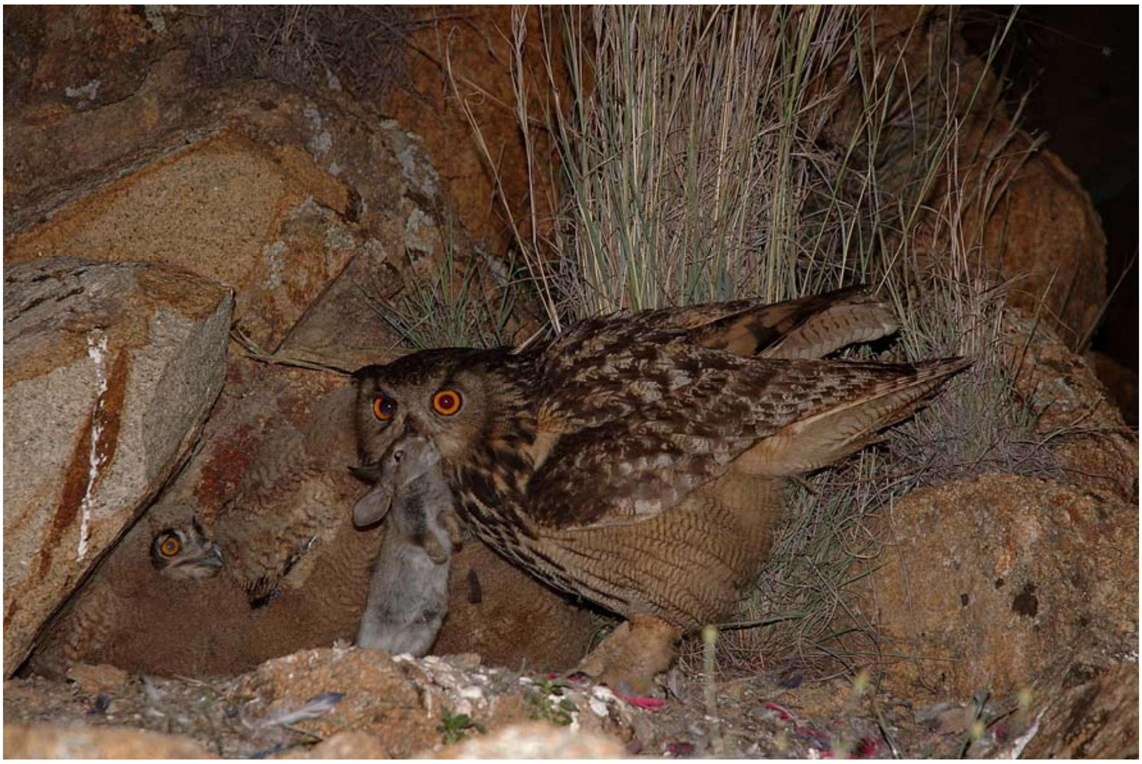

B

Defecation and plucking sites appear during the pre-laying season up until the fledgling period, and peak during the incubation and nestling periods. In these photos, you can see how a faecal mark appears on one of the rocks of the nest cavity during the middle of the nestling period.

S4: TEMPORAL PATTERN OF THE APPEARANCE OF DEFECATION SITES

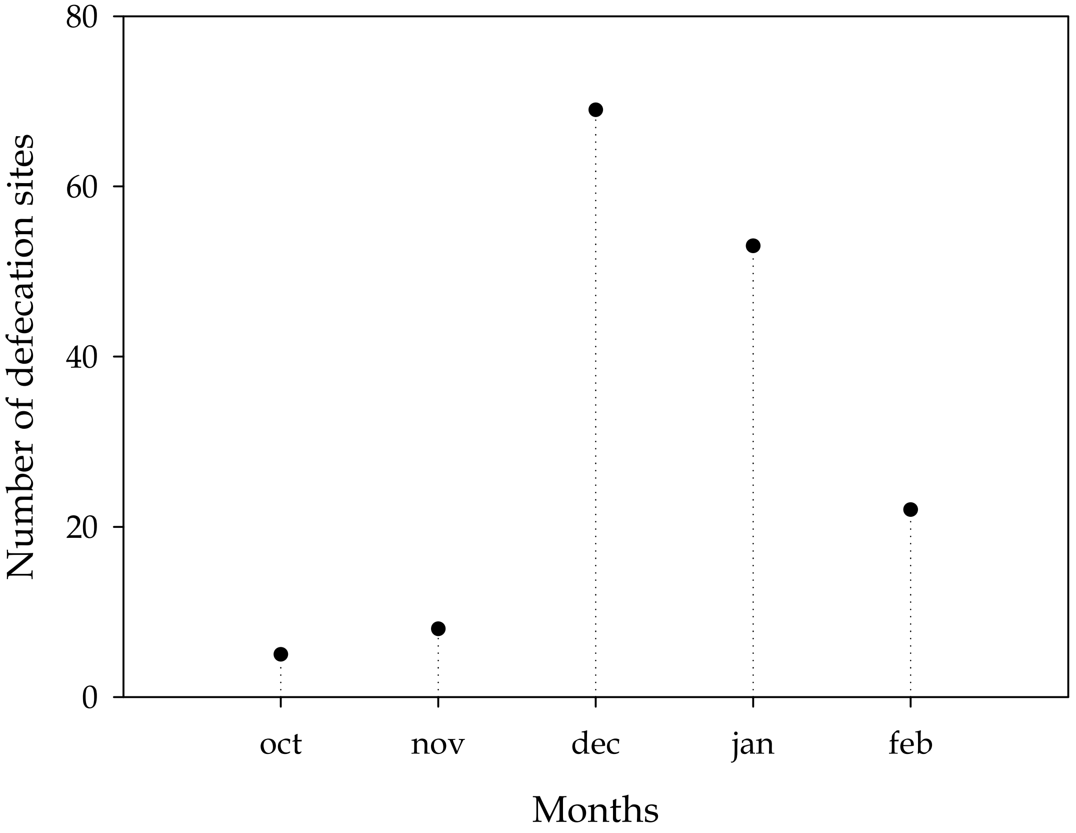

Temporal pattern of the appearance of defecation sites within the 9 home ranges. The number of posts marked by faeces (n = 194) increases during the pre-laying period (October - December in the study area), and peaks just before the month in which most of the females start incubating (January).
