# Supplementary material for: Owls May Use Faeces and Prey Feathers to Signal Current Reproduction
Source: PLoS One. 2008 Aug 20;3(8):e3014. doi: 10.1371/journal.pone.0003014 (PMC2507733; doi:10.1371/journal.pone.0003014)
Supplement: Figure S5 — Some examples of preferential use of darkest substrates for eagle owl faecal marking. (0.35 MB PDF) [file pone.0003014.s005.pdf]

S5: PATTERNS OF FAECAL MARKS ON DARK SUBSTRATES

Some examples showing that eagle owls preferentially leave faecal marks on the darkest substrates. In A, B and C, owls mark the most prominent and darkest rocks within close proximity to their nest.

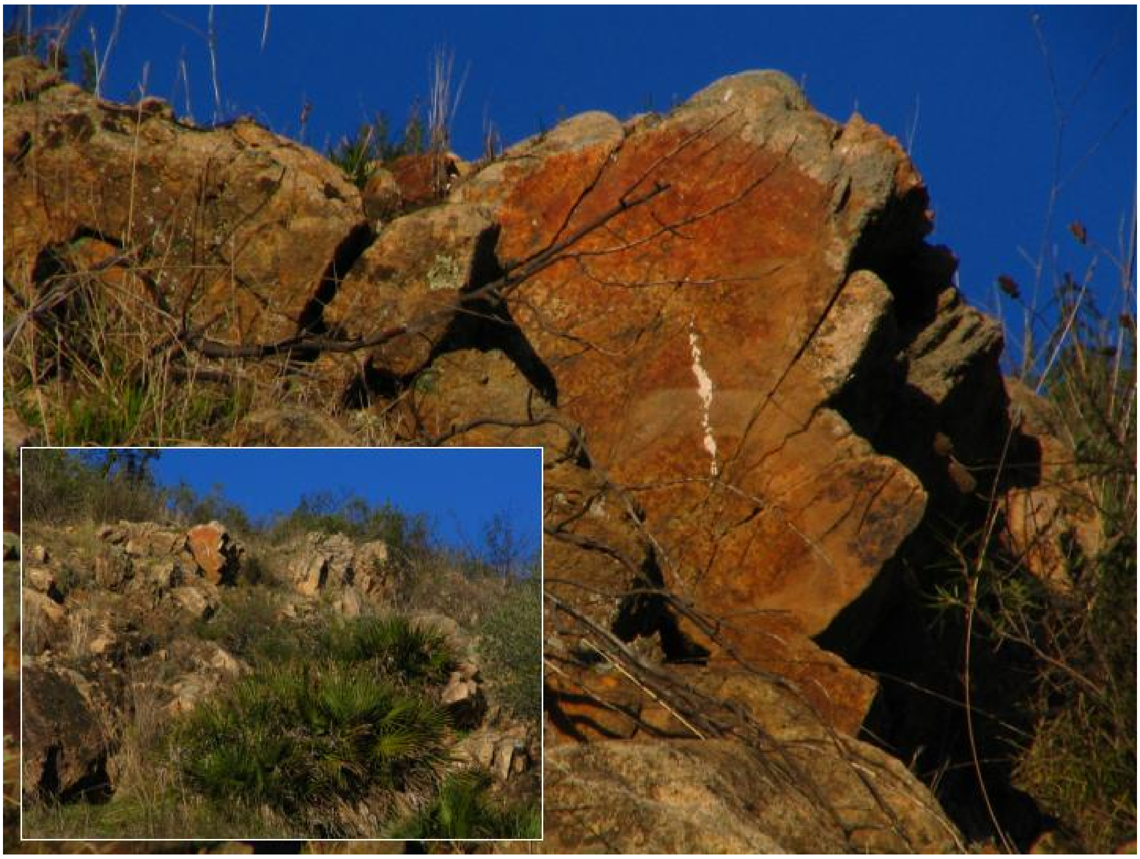

A

S5: PATTERNS OF FAECAL MARKS ON DARK SUBSTRATES

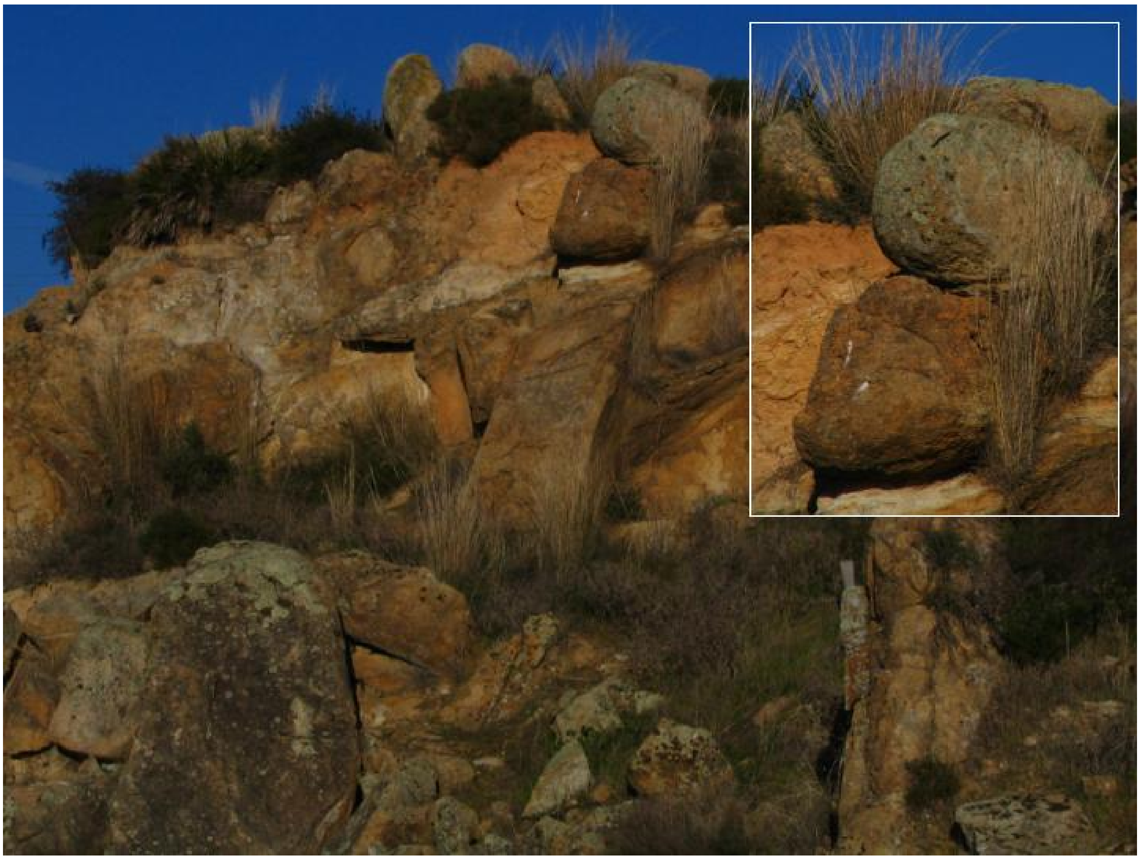

B

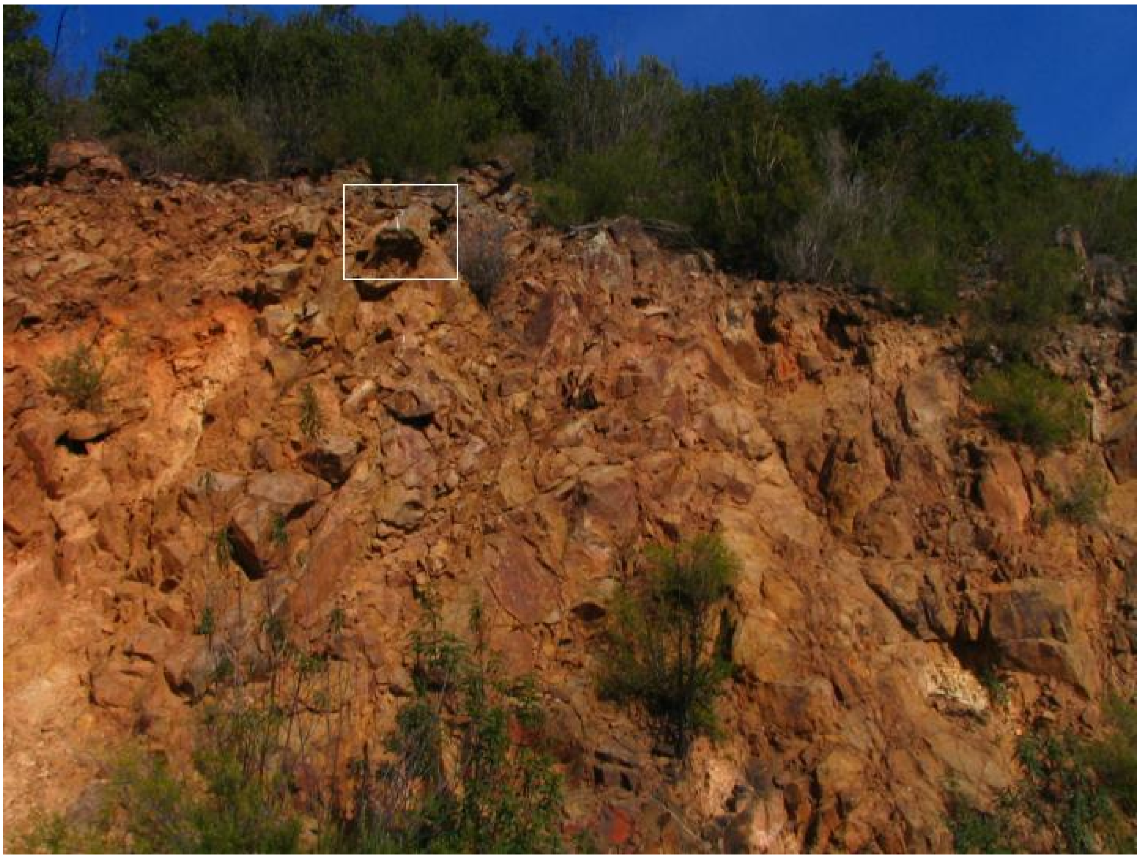

C

S5: PATTERNS OF FAECAL MARKS ON DARK SUBSTRATES

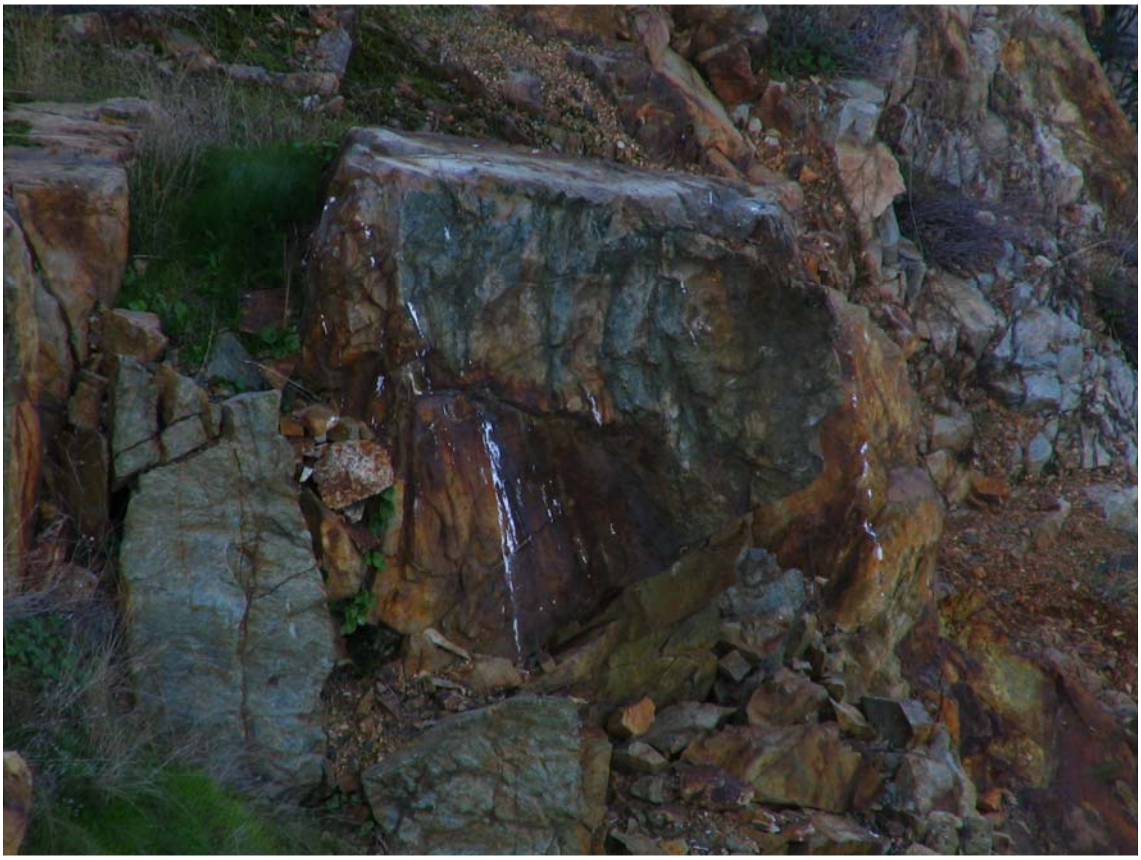

D

D shows a frequent marking pattern that uses the darkest section of the substrate.
