# Supplementary material for: Owls May Use Faeces and Prey Feathers to Signal Current Reproduction
Source: PLoS One. 2008 Aug 20;3(8):e3014. doi: 10.1371/journal.pone.0003014 (PMC2507733; doi:10.1371/journal.pone.0003014)
Supplement: Figure S6 — Both faecal marks and plucking sites are located in positions with increased conspicuousness, such as dominant places and the highest points of valley slopes. Some marks also appear at the entrance of the valley in which the nest is located. (5.80 MB PDF) [file pone.0003014.s006.pdf]

Both faecal marks (from A to D and from F to L) and plucking sites (E) are placed on positions increasing their conspicuousness, like dominant places (A, B, D, E and K) and the highest points of valley slopes (C, H, I and J).

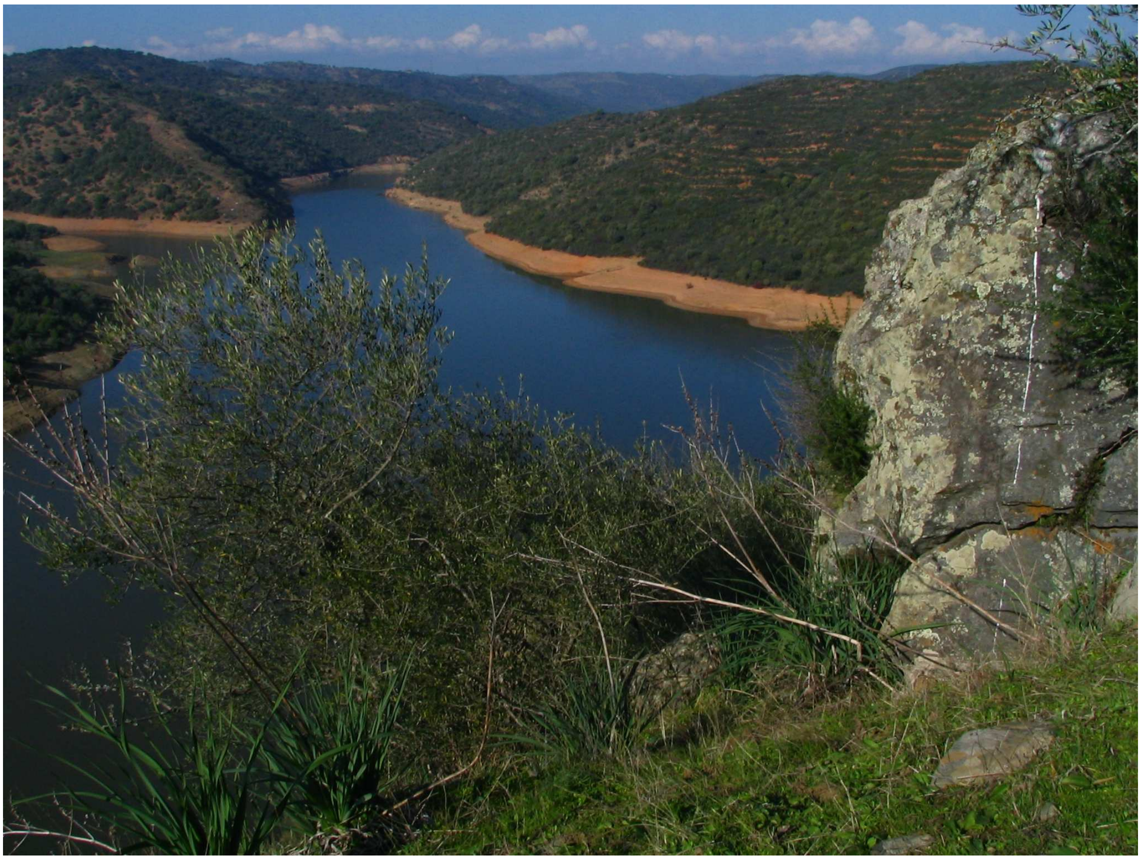

A

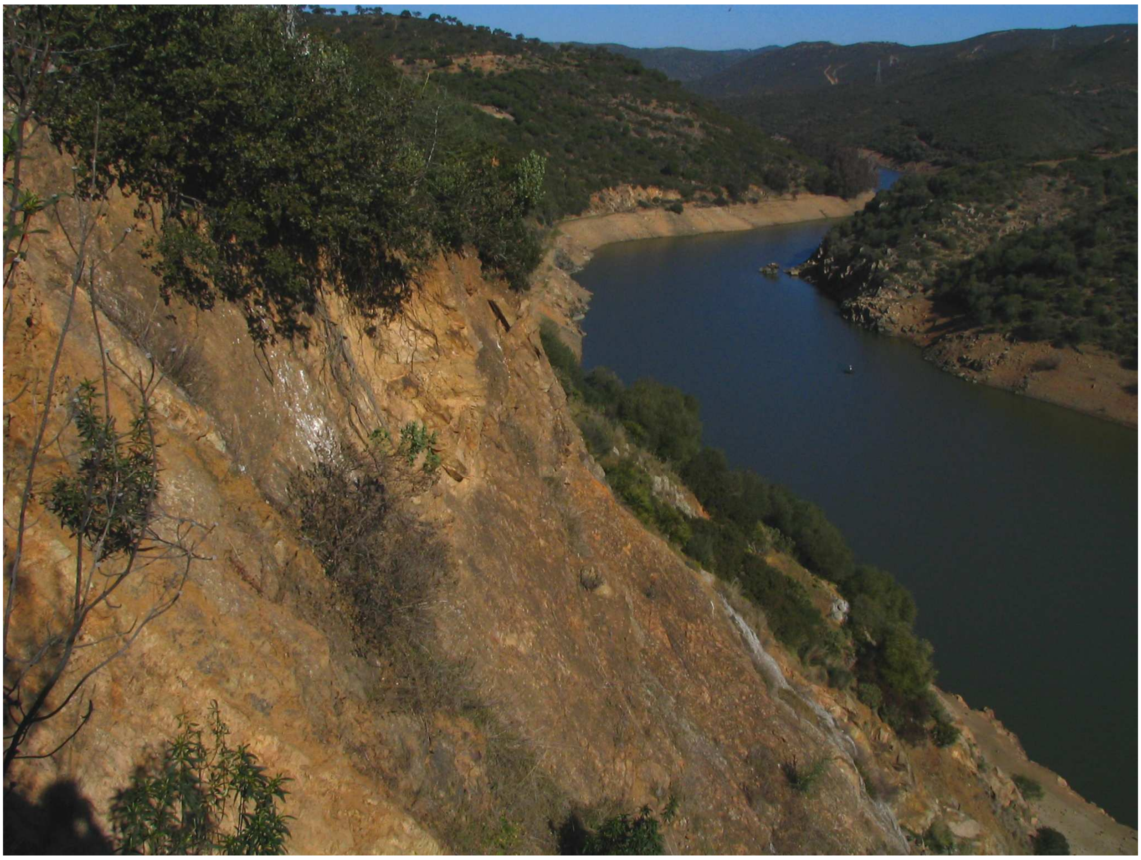
B

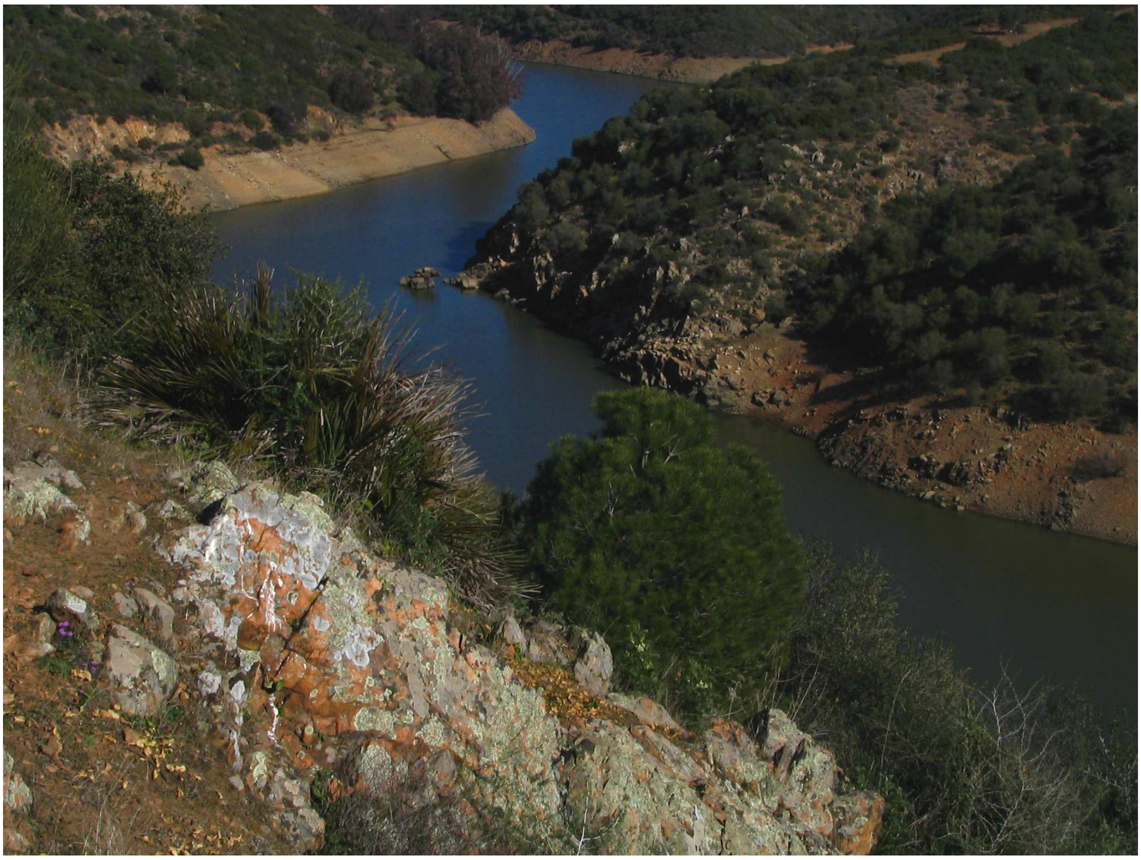
C

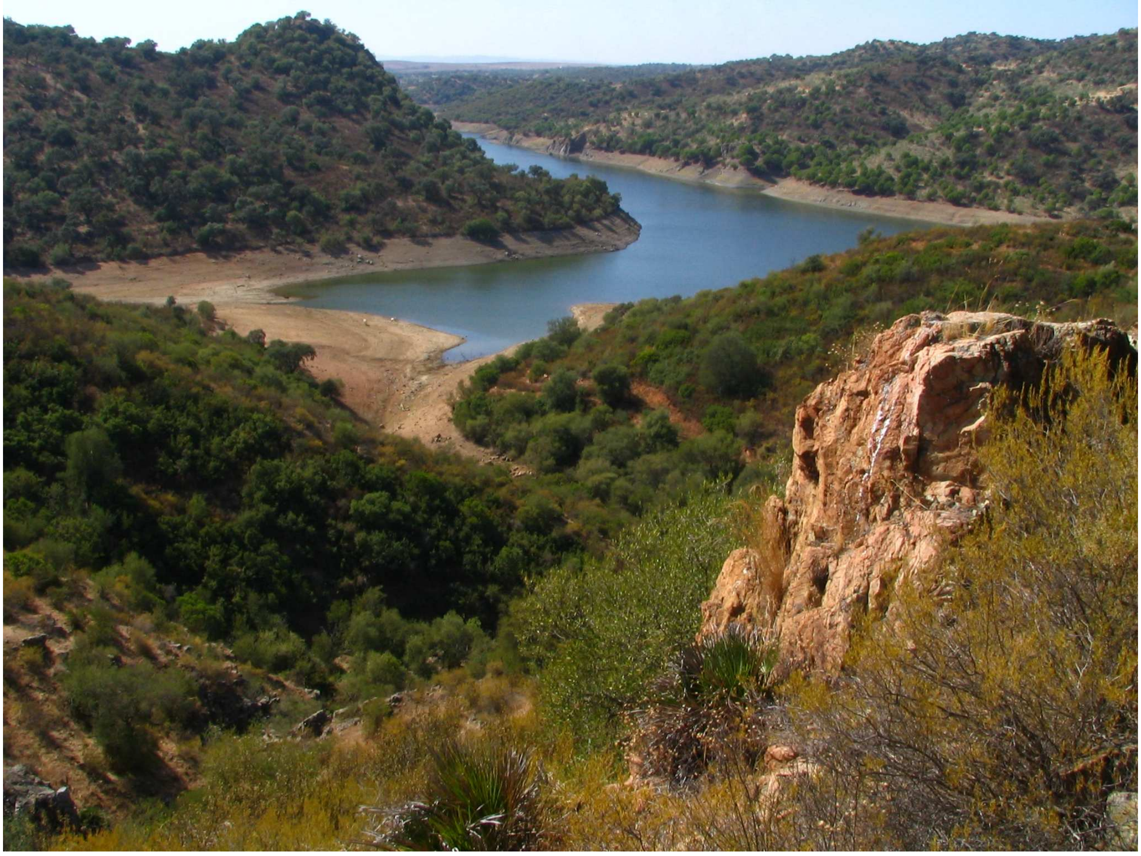

D

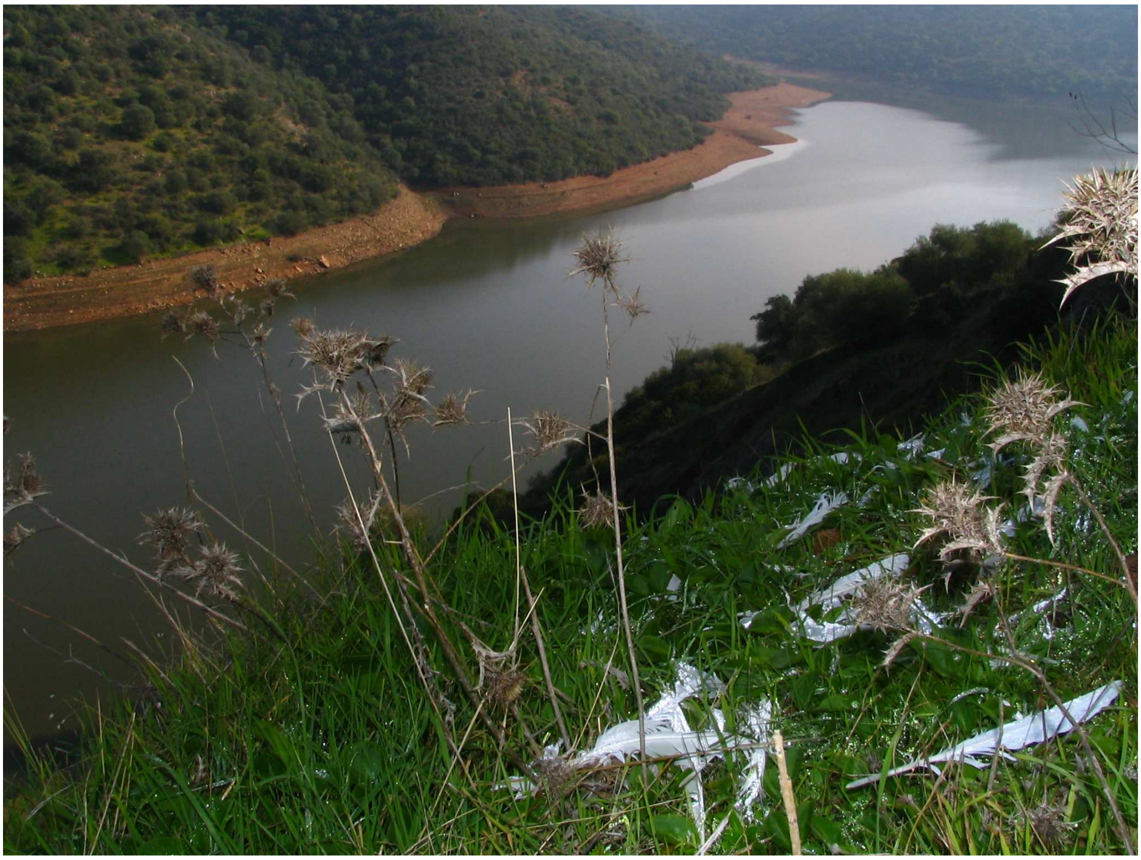

E

Some marks also appear at the entrance of the valley in which the nest is located (F and G). That is, marking is always done on strategic positions giving such long lasting, visual signals easily detectable from the nearest neighbour territories or moving individuals (e.g. floaters).

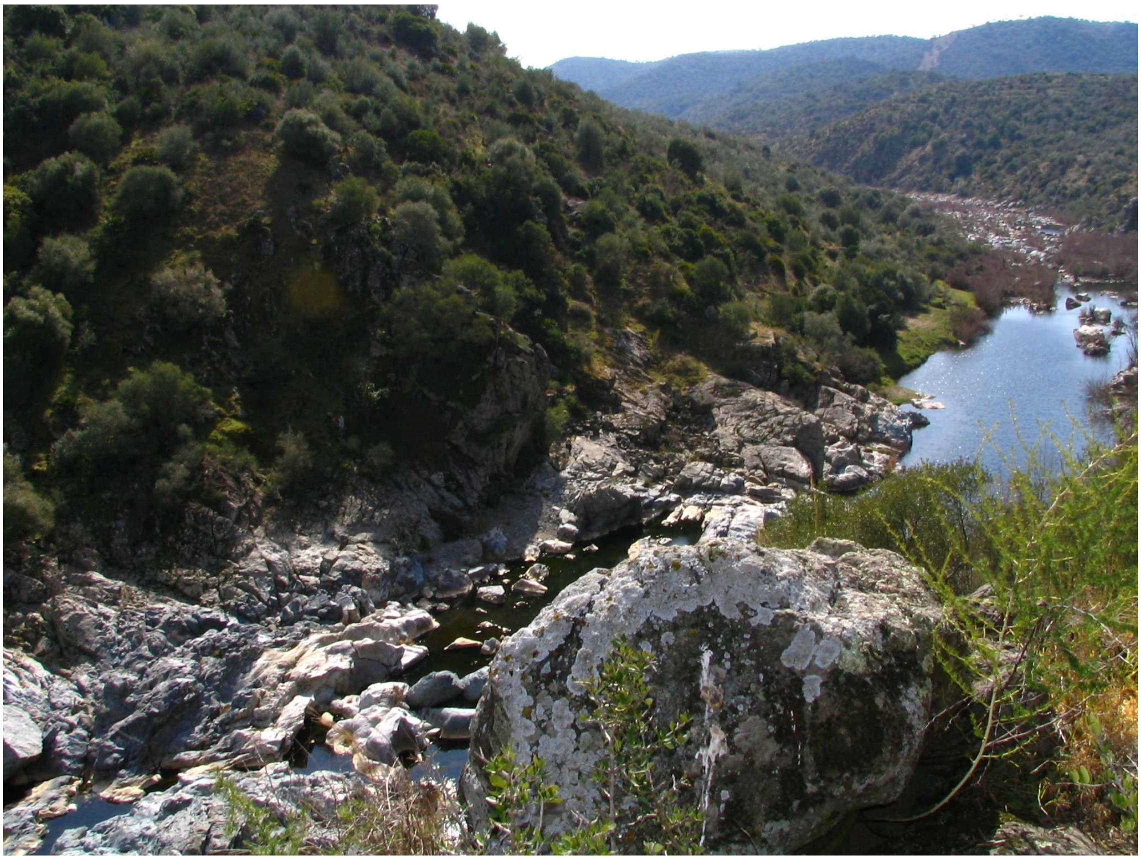

F

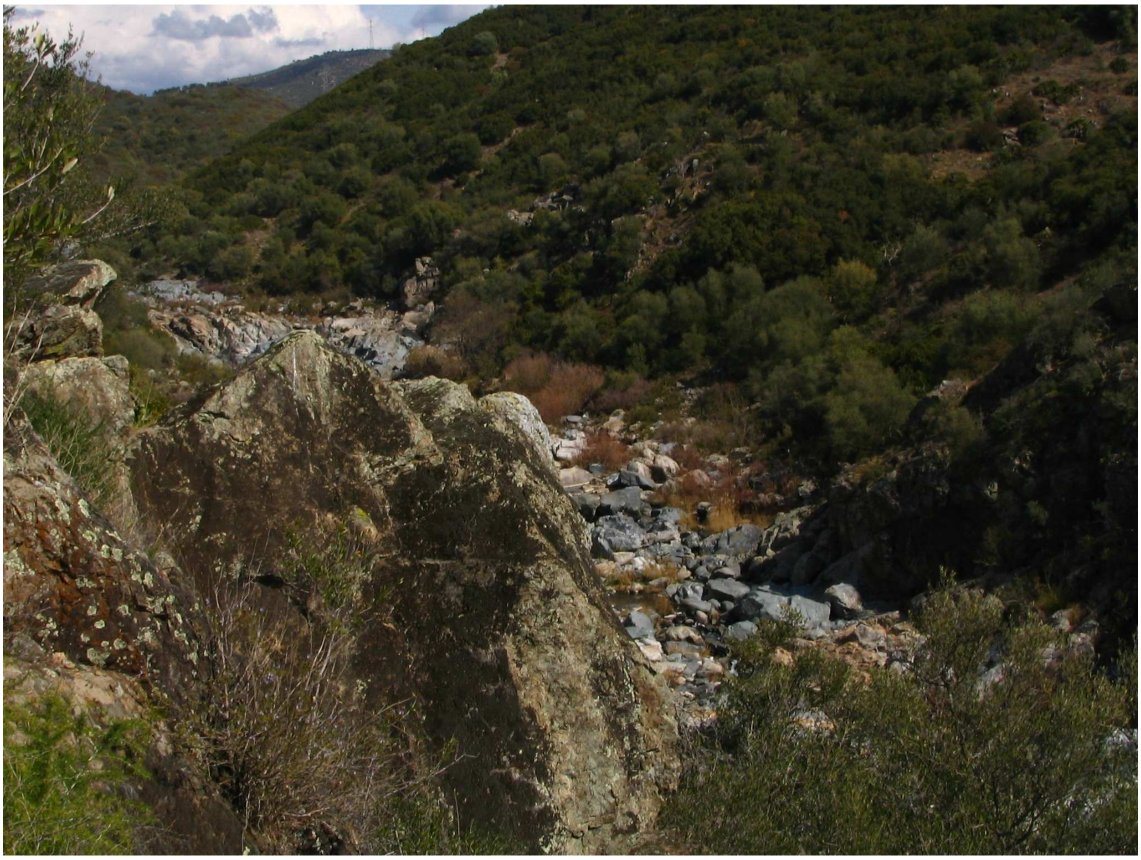
G

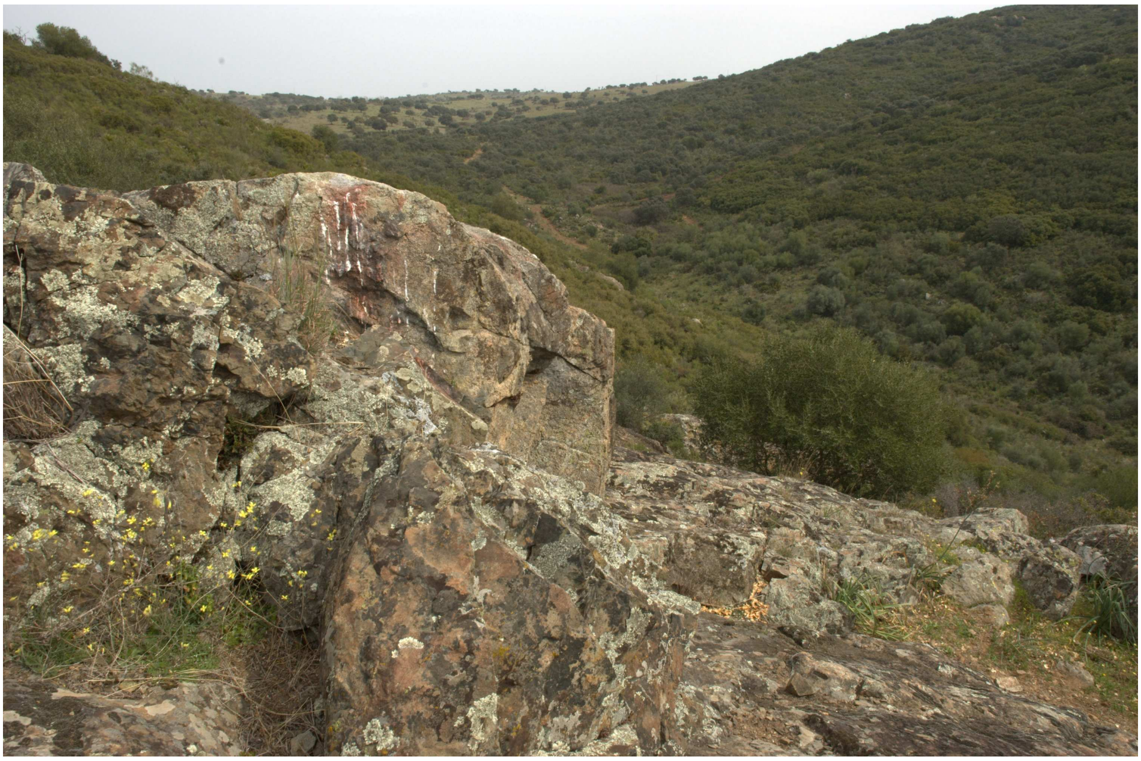
H

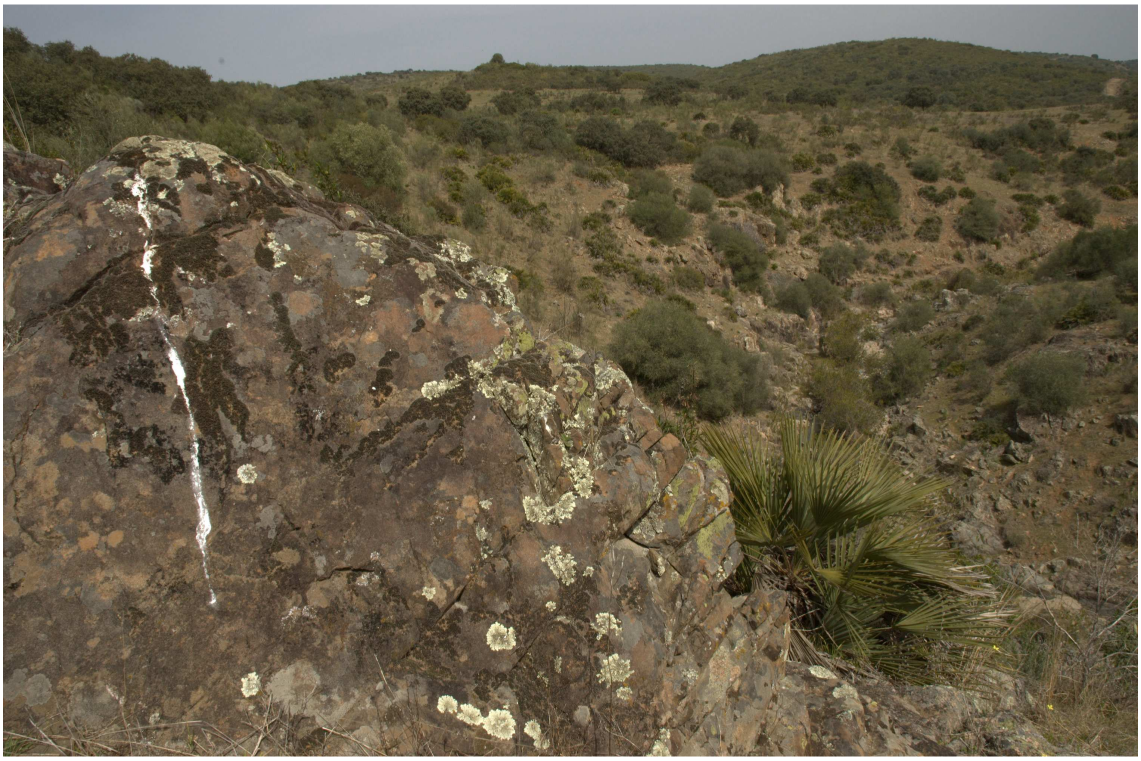
I

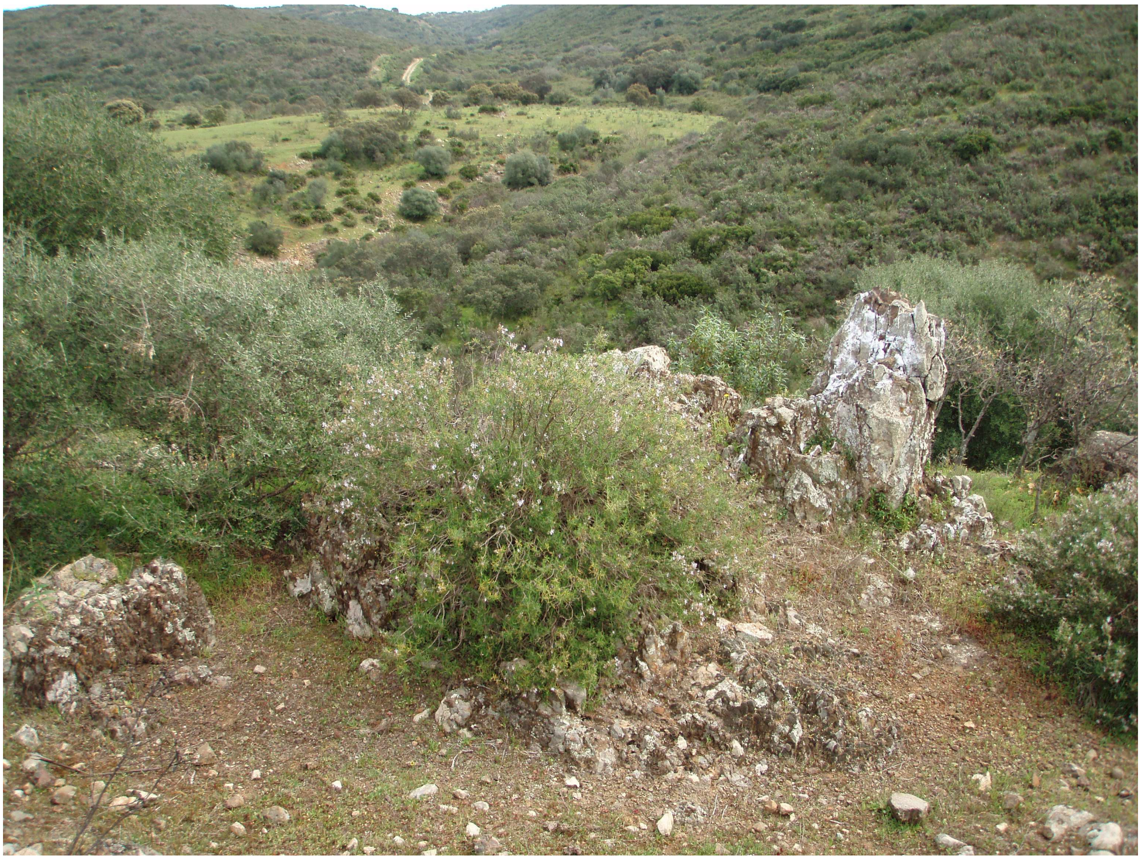
J

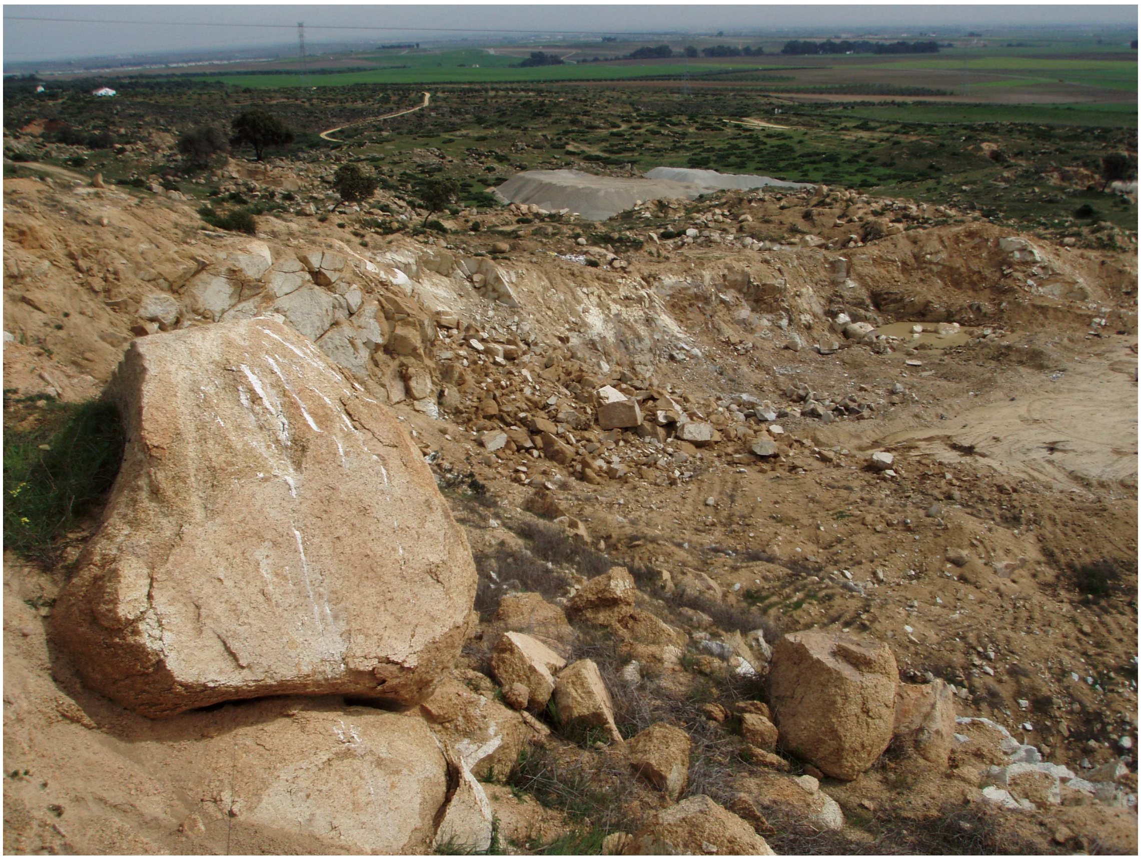
K

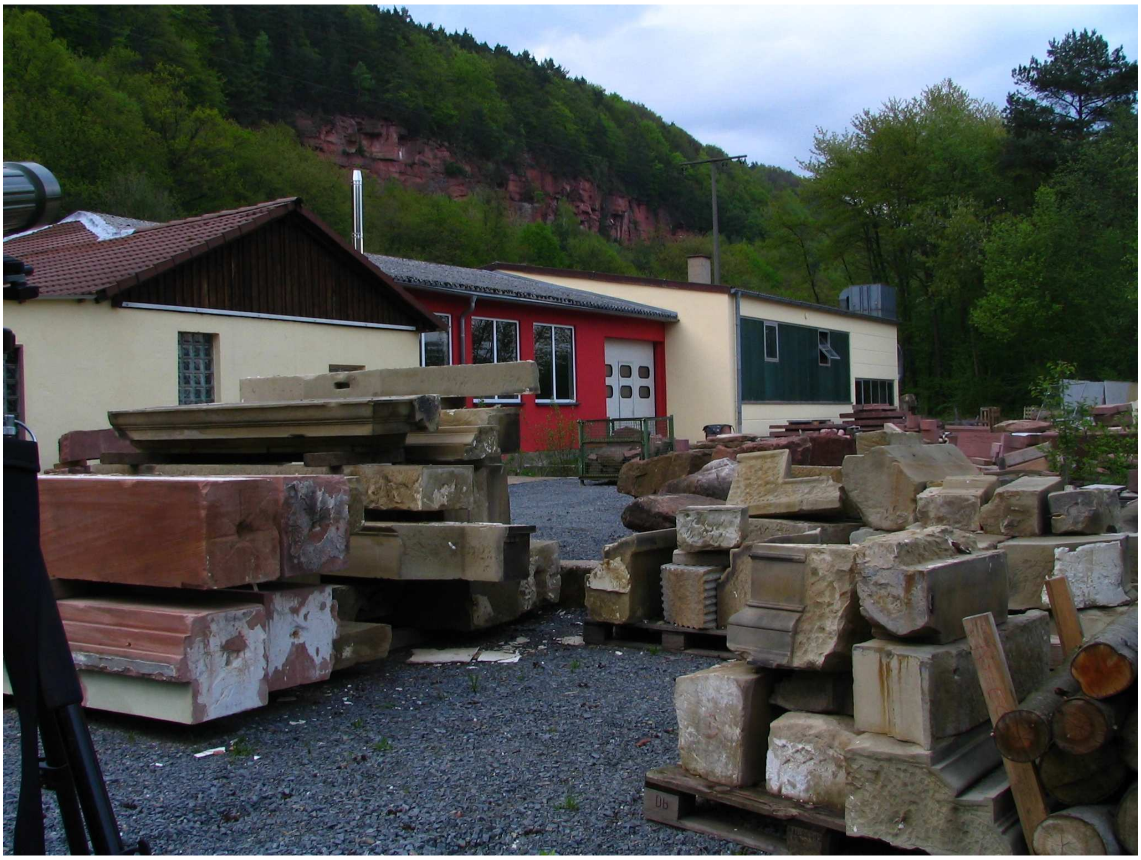

L

The extreme visibility of the white faeces, due to both their contrast on dark surfaces and dominant position, is appreciable in L.
