# Supplementary material for: Owls May Use Faeces and Prey Feathers to Signal Current Reproduction
Source: PLoS One. 2008 Aug 20;3(8):e3014. doi: 10.1371/journal.pone.0003014 (PMC2507733; doi:10.1371/journal.pone.0003014)
Supplement: Figure S7 — Examples of faecal marks being refreshed after we experimentally obscured them with spray paint. Generally, the eagle owls returned to re-mark within one to two nights of the experimental covering. In several cases, faeces were scattered at exactly the same position that had been previously marked. (0.89 MB PDF) [file pone.0003014.s007.pdf]

S7: REFRESHING OF FAECAL MARKS

Examples of faecal marks being refreshed after we experimentally covered them with spray paint. Generally, eagle owls come back to re-mark the site within one or two nights. Notably, in several cases (A, B, D, E, F, H and J) faeces are scattered at exactly the same position occupied by the obscured markings.

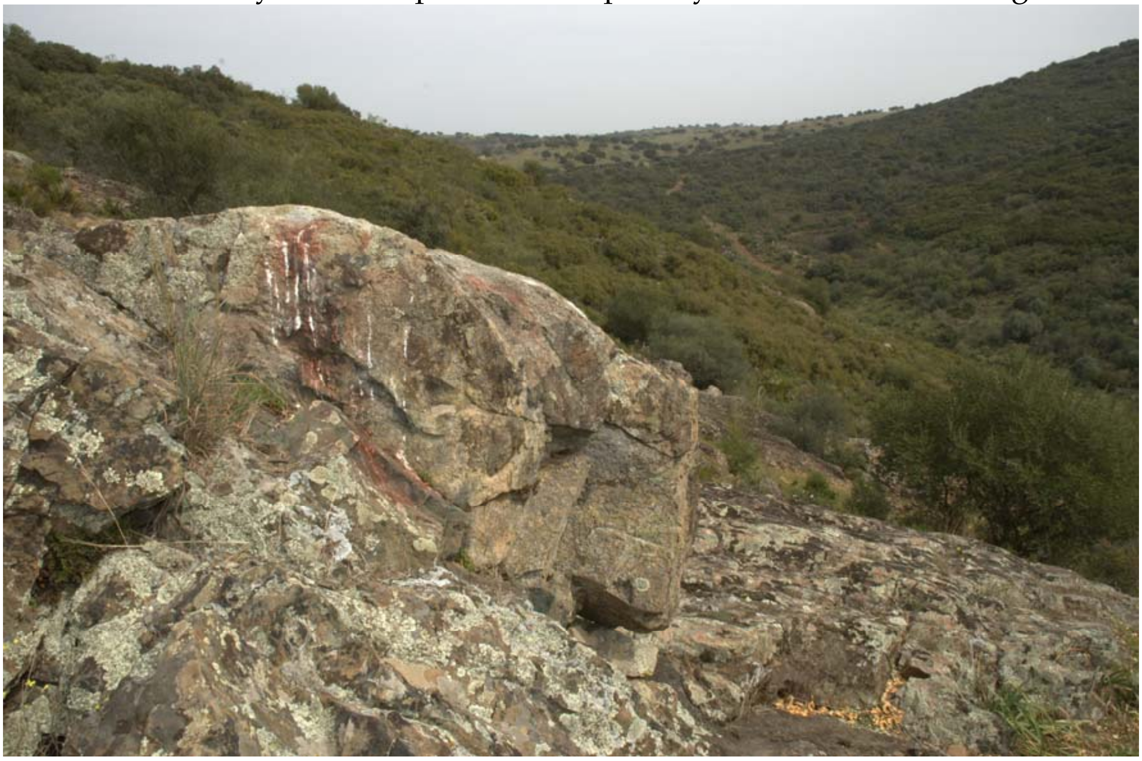

A

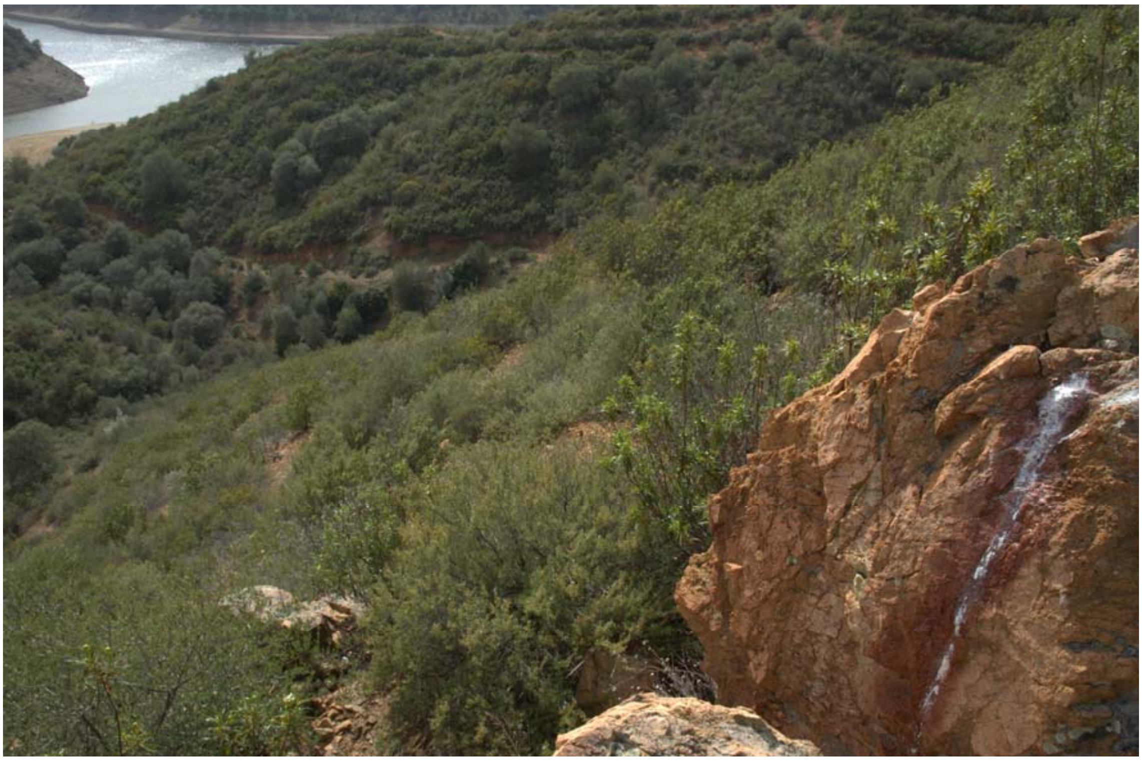

B

S7: REFRESHING OF FAECAL MARKS

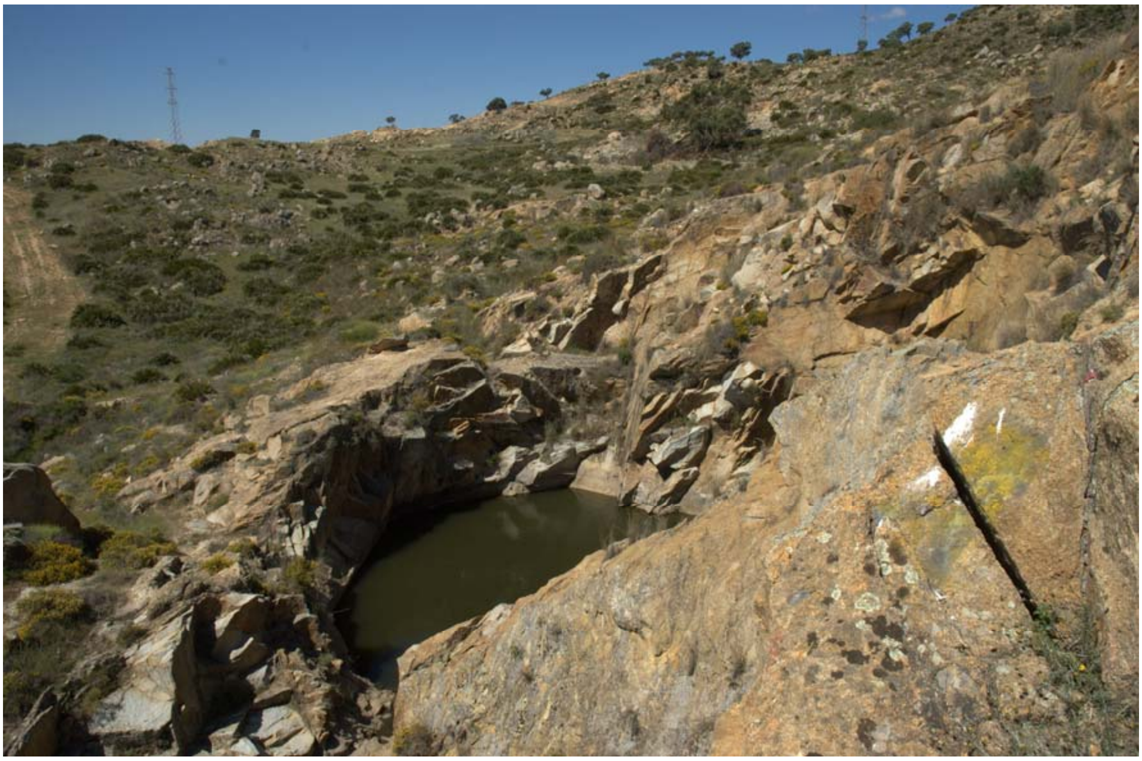

C

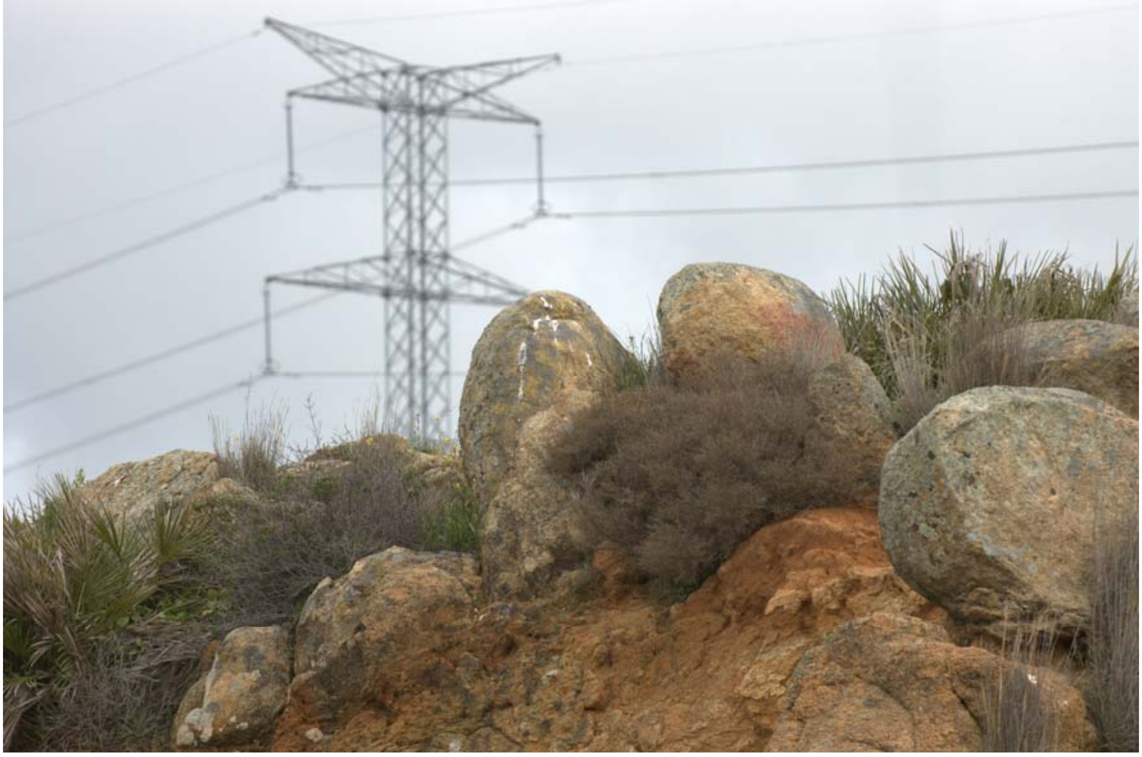

D

S7: REFRESHING OF FAECAL MARKS

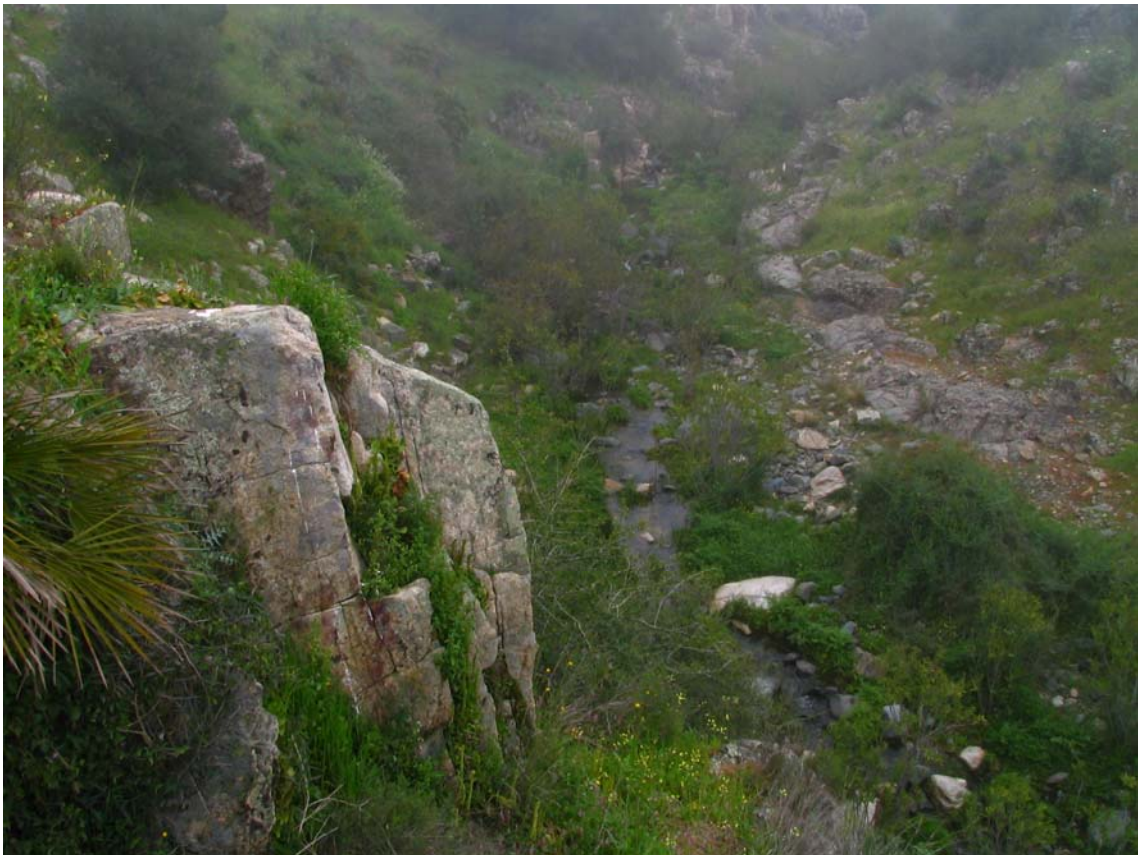

E

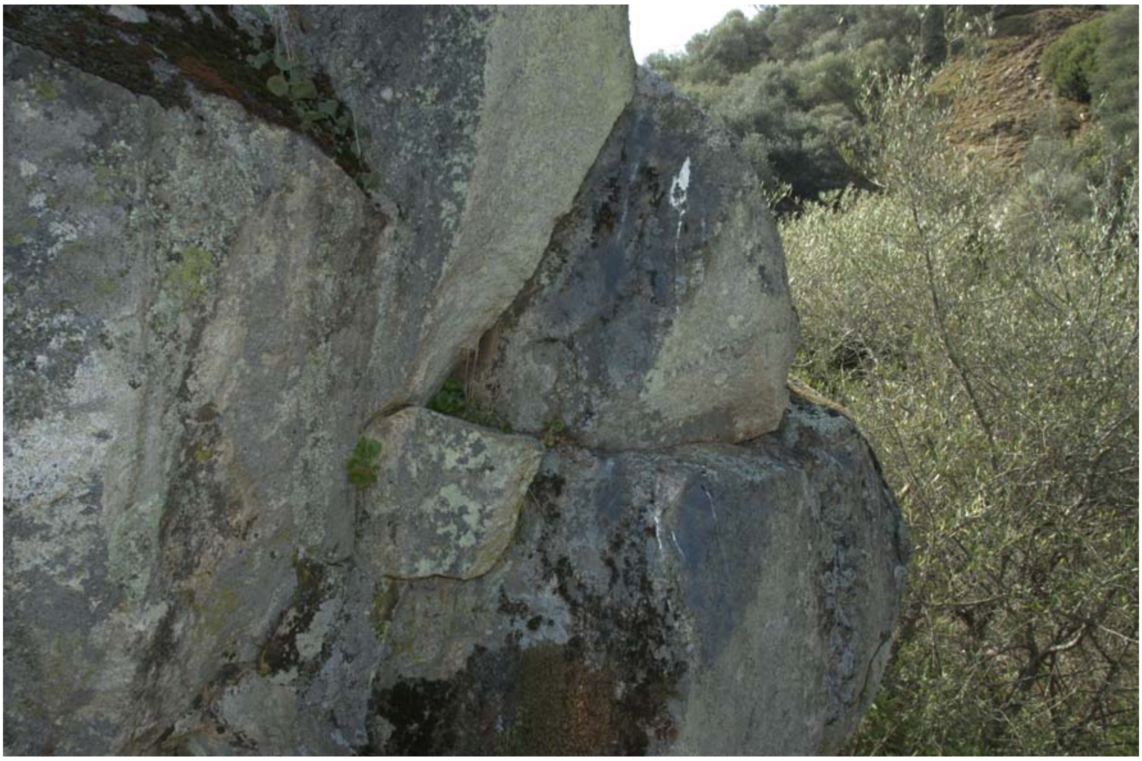

F

S7: REFRESHING OF FAECAL MARKS

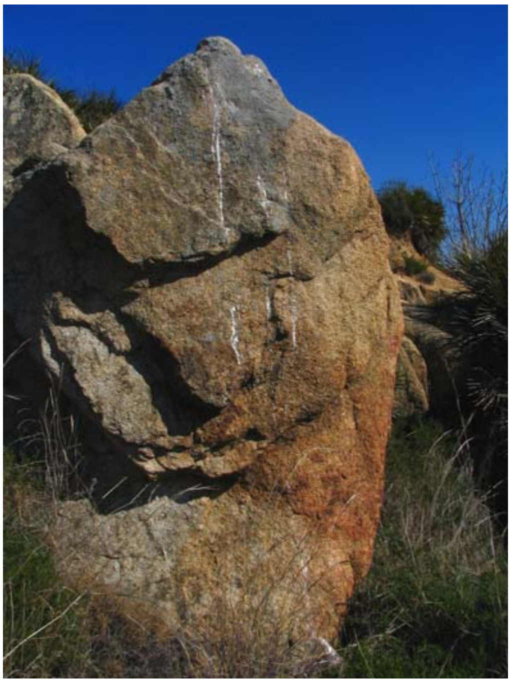

G

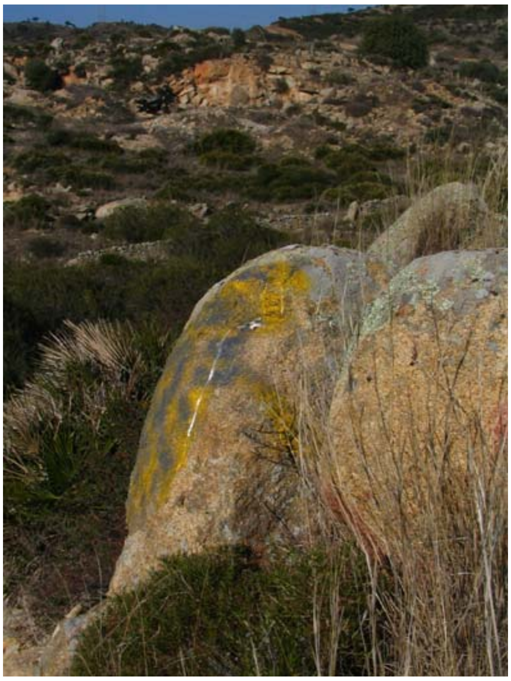

H

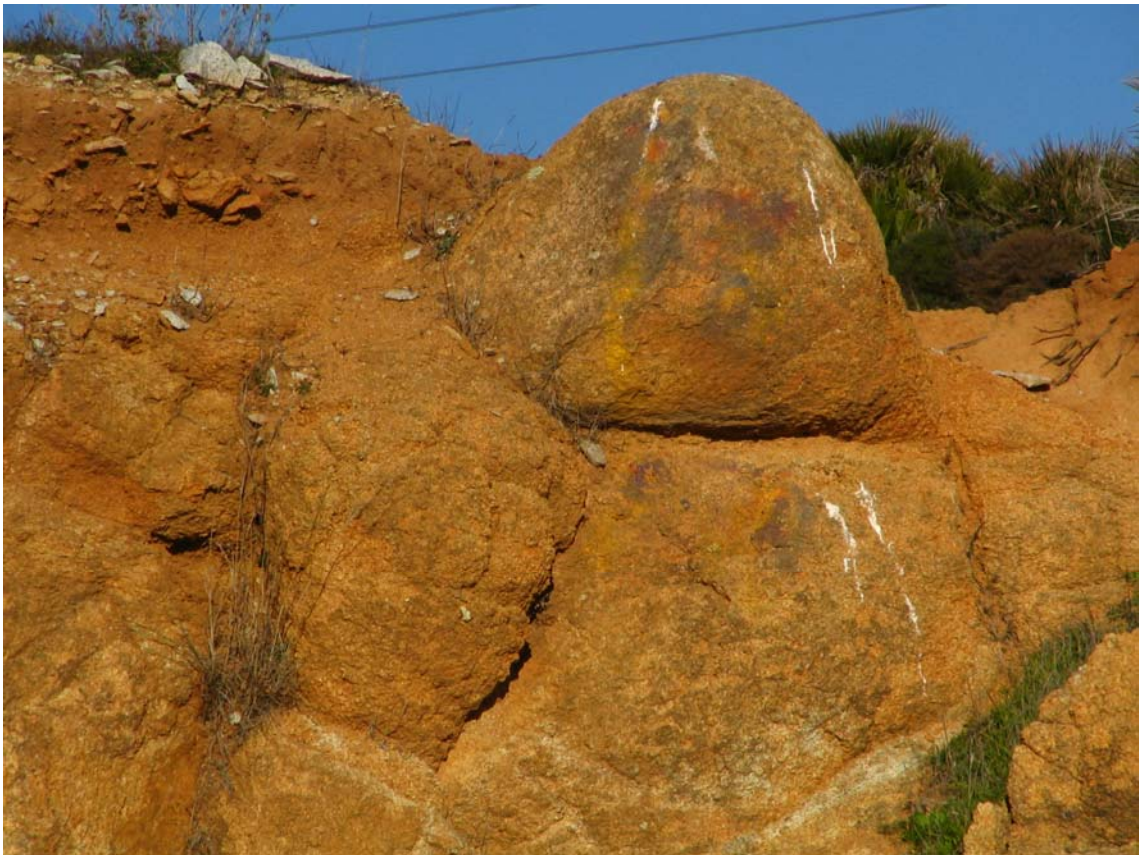

I

S7: REFRESHING OF FAECAL MARKS

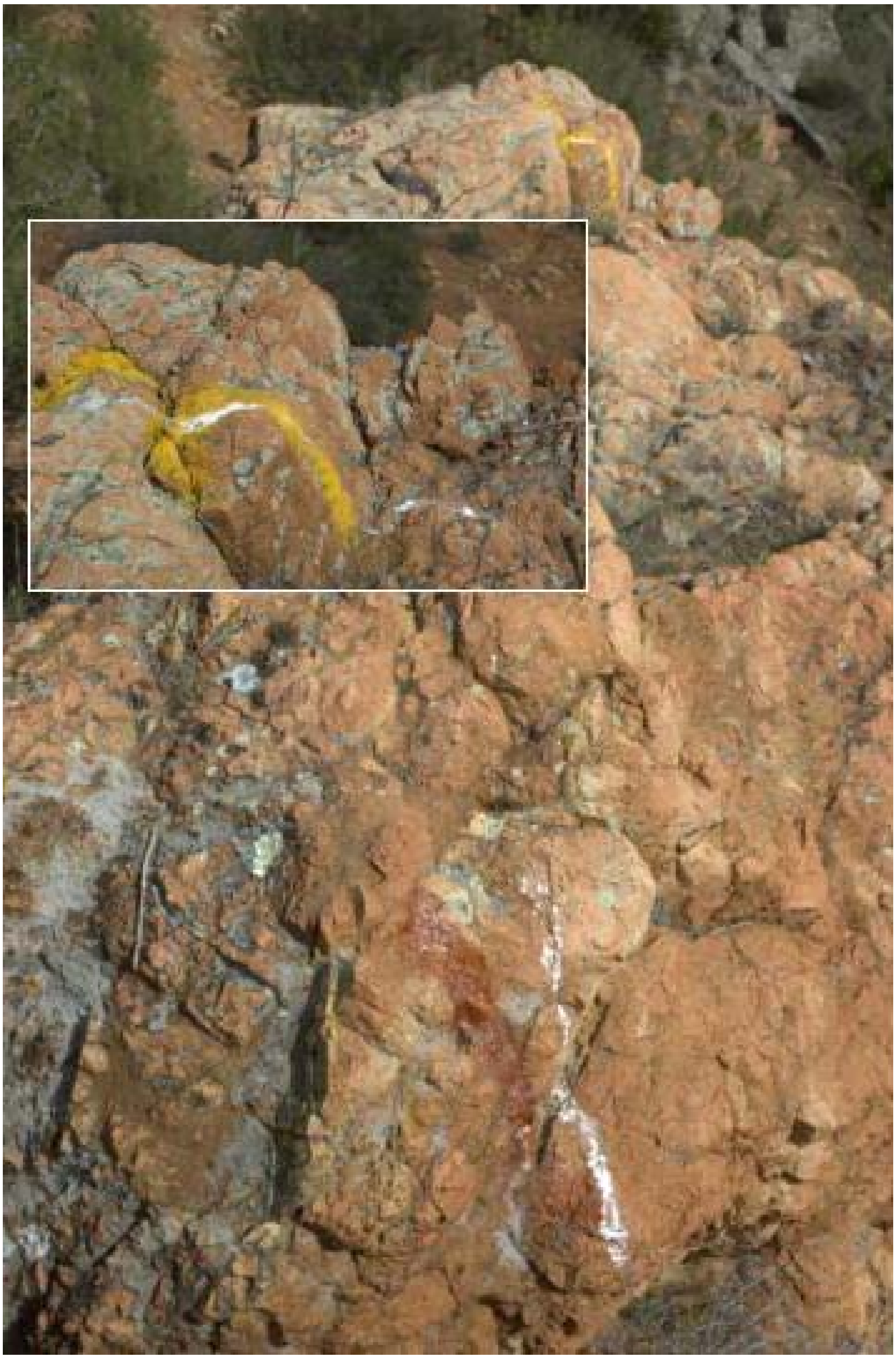

J
