# Supplementary material for: Owls May Use Faeces and Prey Feathers to Signal Current Reproduction
Source: PLoS One. 2008 Aug 20;3(8):e3014. doi: 10.1371/journal.pone.0003014 (PMC2507733; doi:10.1371/journal.pone.0003014)
Supplement: Figure S8 — Pictures showing the significant contrast between the bright feathers and the dark surface of the plucking site. All the prey species on plucking sites were birds with highly visible feathers. (0.59 MB PDF) [file pone.0003014.s008.pdf]

As seen for faecal marks, plucking sites are very close to the nests (circled in A and B). The pictures also show the great contrast between the bright feathers and the dark surface of the plucking site.

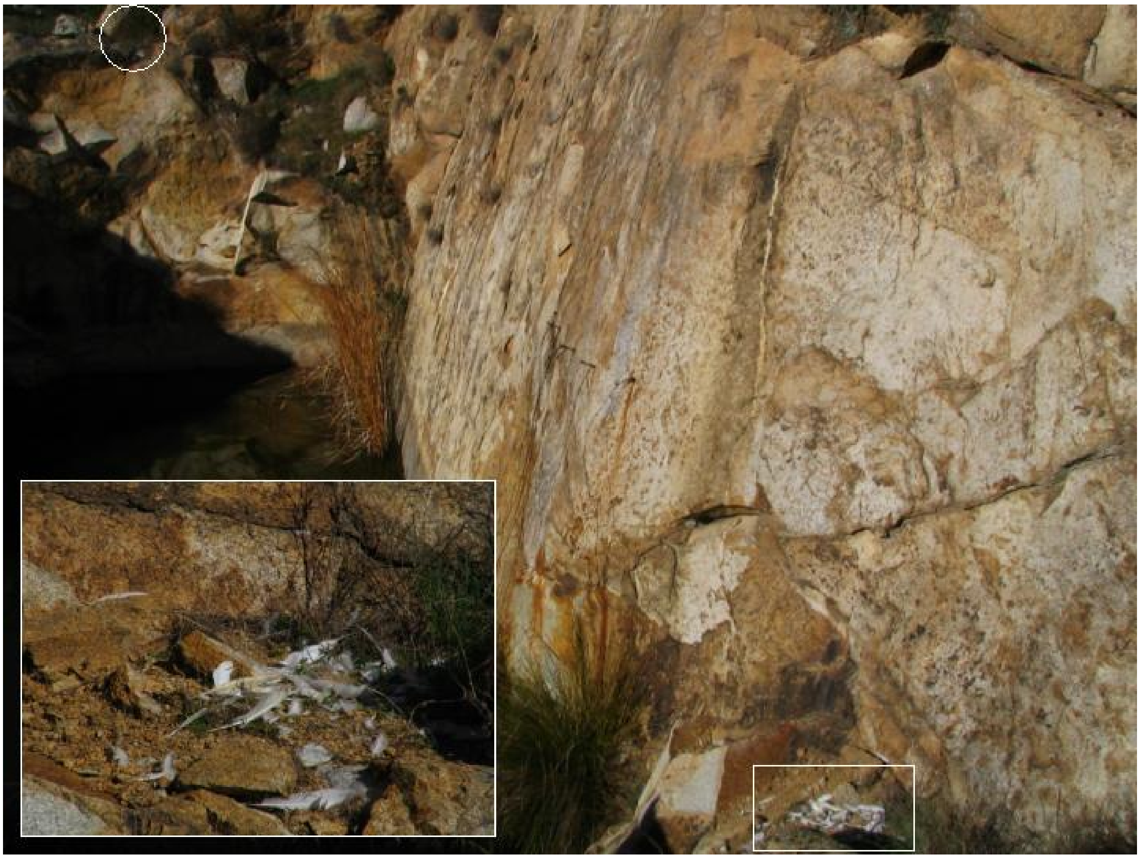

A

All prey species found on the plucking sites are birds with highly visible feathers, namely the azure-winged magpie (*Cyanopica cyanus*, n = 3), barn owl (*Tyto alba* = 2), *Larus* spp. (n = 1), little egret (*Egretta garzetta*, n = 3), red-legged partridge (*Alectoris rufa*, n = 14), short-eared owl (*Asio flammeus*, n = 1) and wood pigeon (*Columba palumbus*, n = 17).

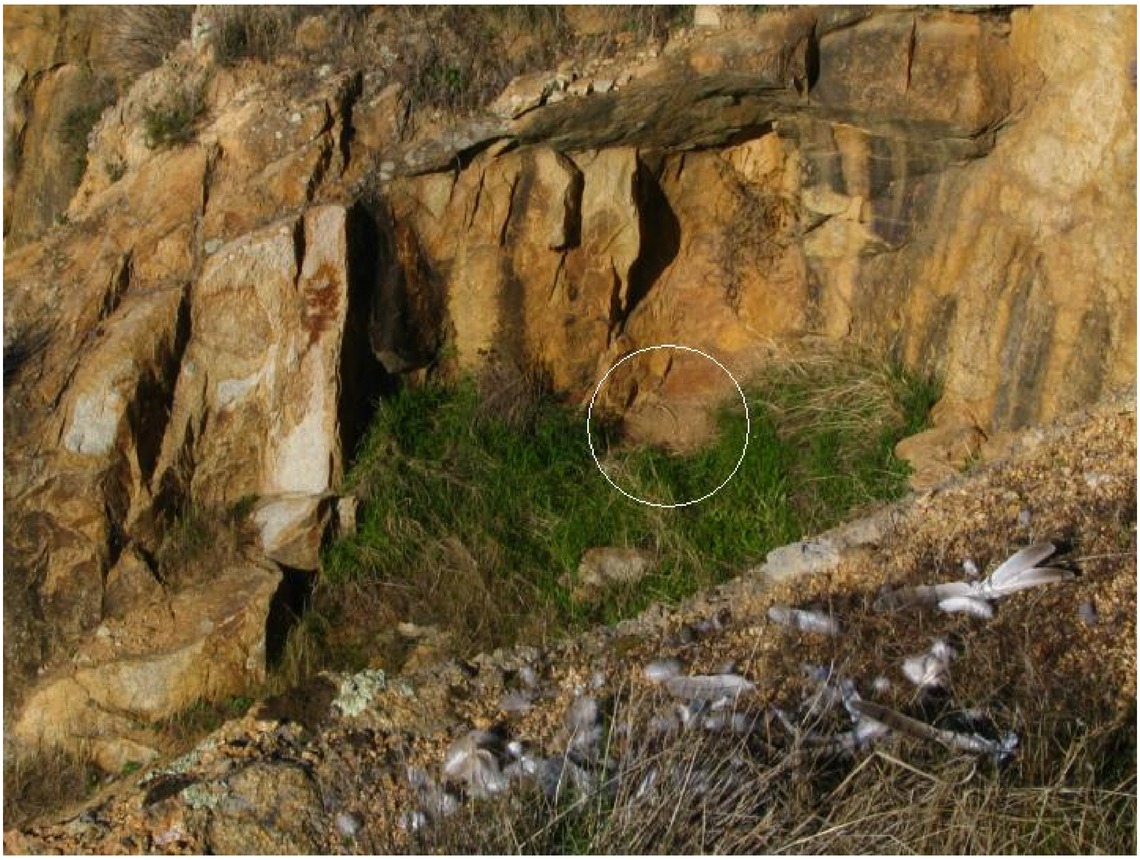

B

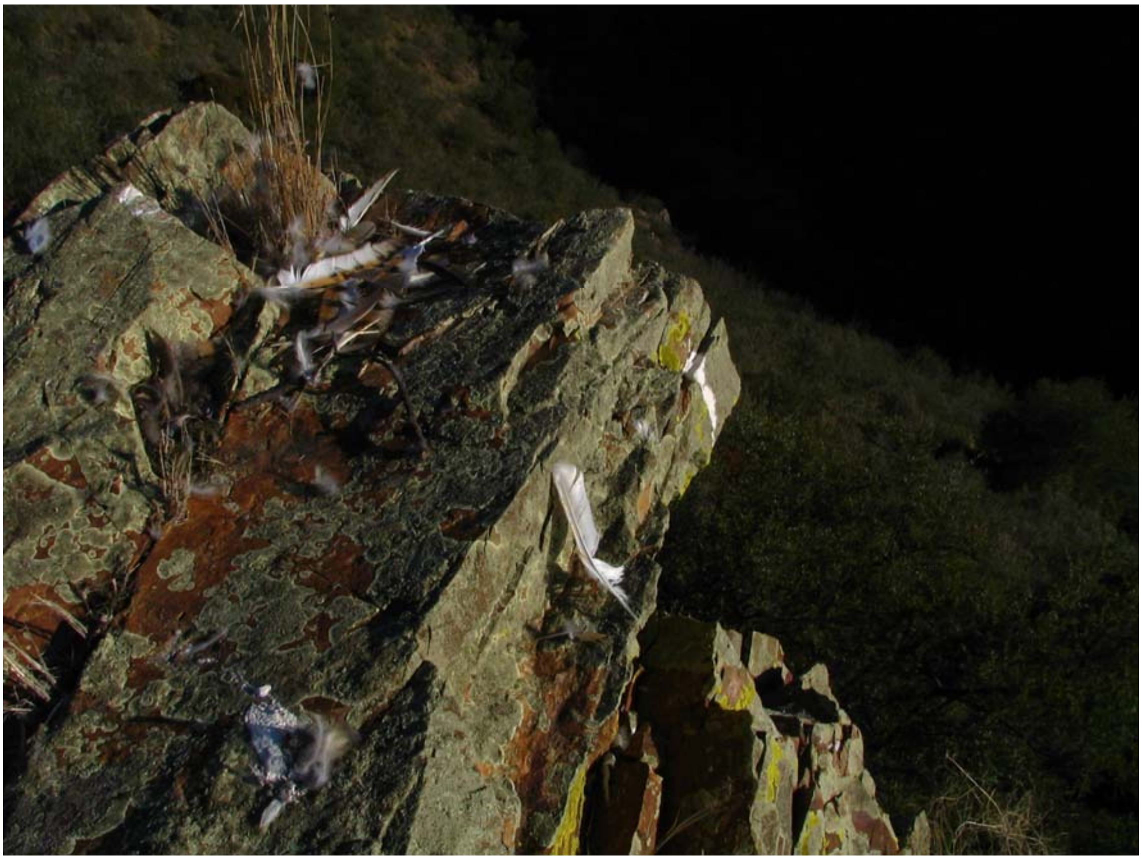

C

Faeces and prey remains may be used to mark the same post.

S8: FEATURES OF PLUCKING SITES

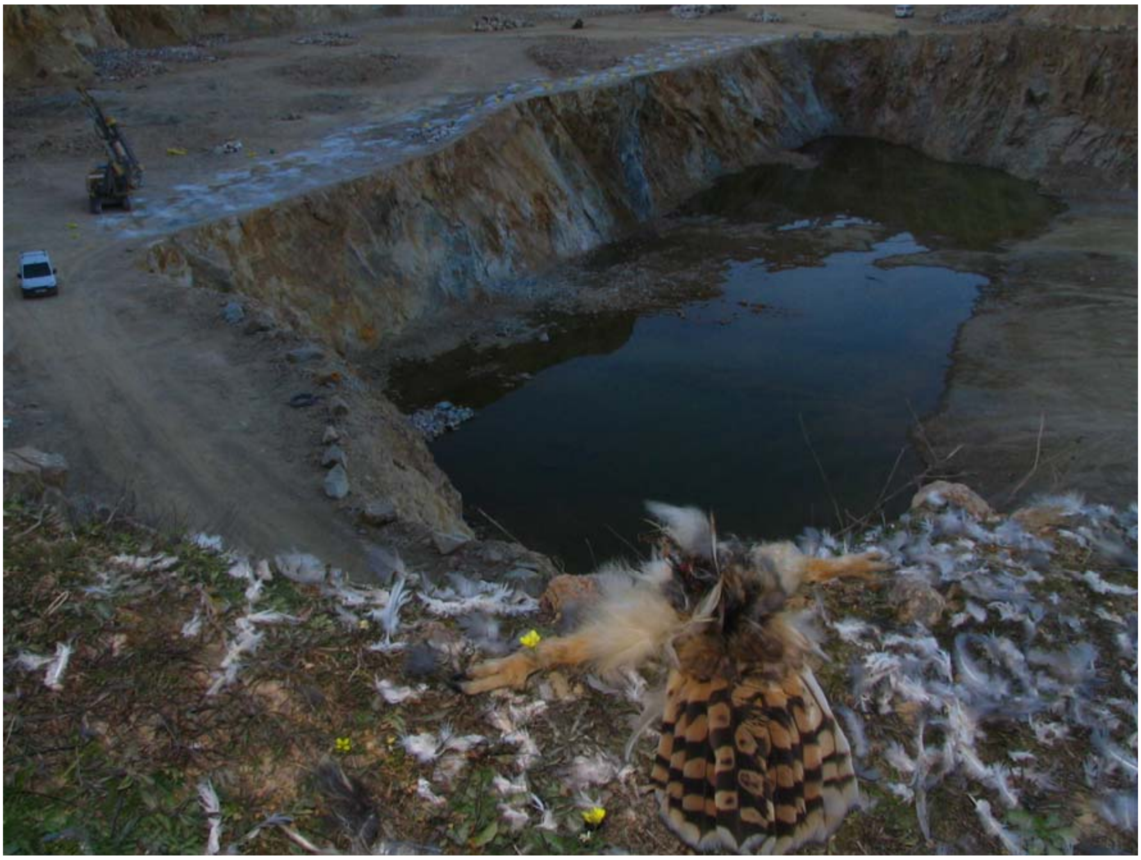

D

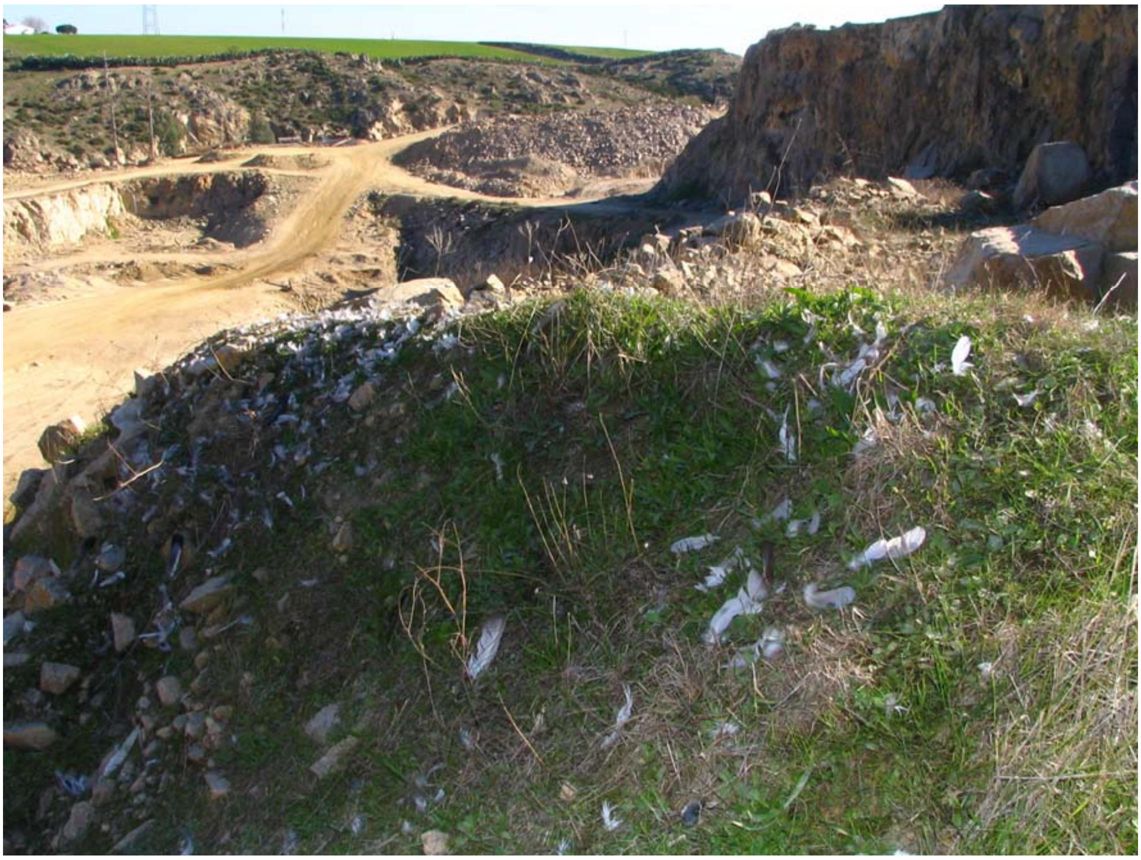

E

S8: FEATURES OF PLUCKING SITES

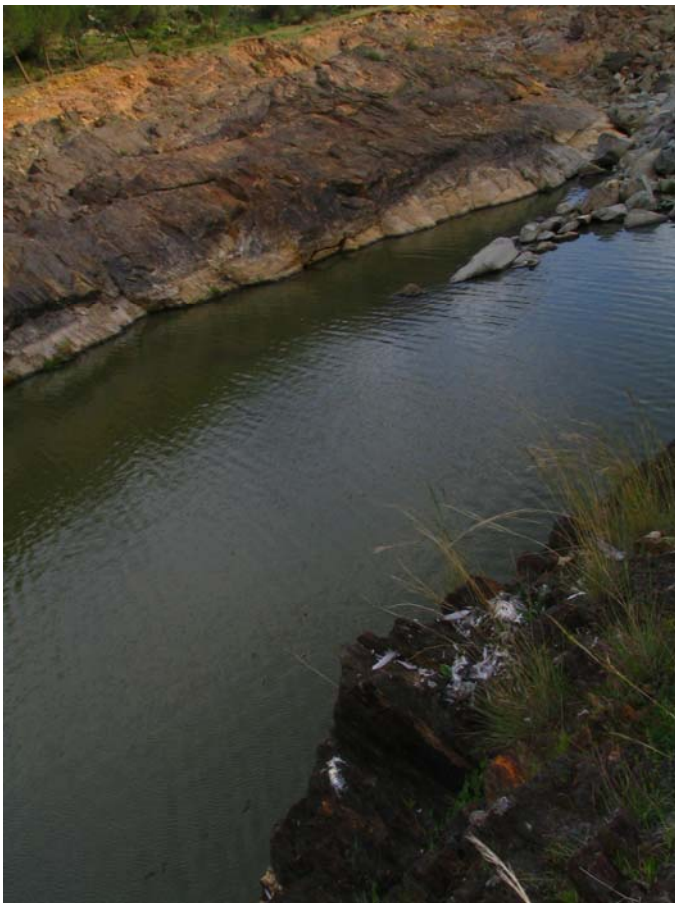

F
