# Supplementary material for: Owls May Use Faeces and Prey Feathers to Signal Current Reproduction
Source: PLoS One. 2008 Aug 20;3(8):e3014. doi: 10.1371/journal.pone.0003014 (PMC2507733; doi:10.1371/journal.pone.0003014)
Supplement: Figure S9 — Some faecal marks were only visible from the nest, not the surroundings. In such a context, they could act to signal reproductive status between the male and female of the breeding pair. (0.24 MB PDF) [file pone.0003014.s009.pdf]

S9: FAECAL MARKS AND MATE-MATE COMMUNICATION

Some faecal marks are only visible from the nest (in both examples shown here, the nest is on the cliff just in front of the mark), not the surroundings. Thus, these markings could signal reproductive status in communications between breeding partners (e.g. the location of preferred nest placement).

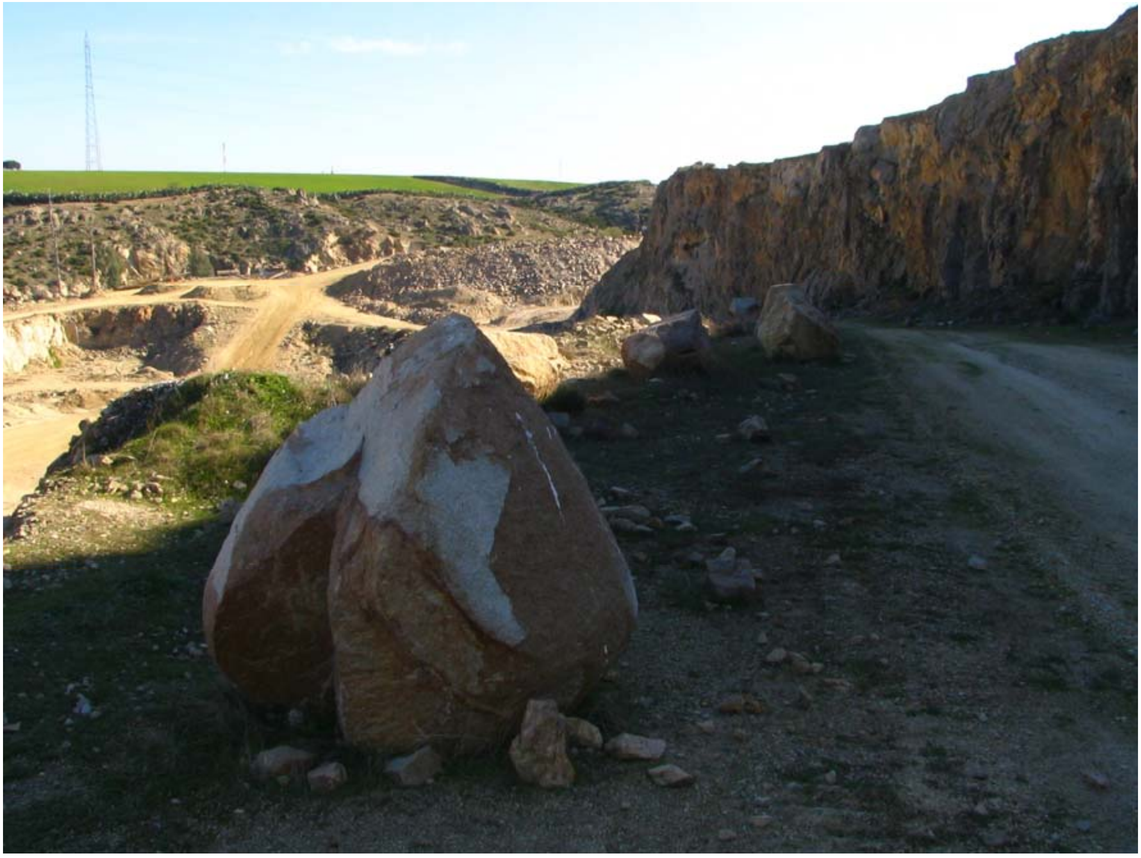

A

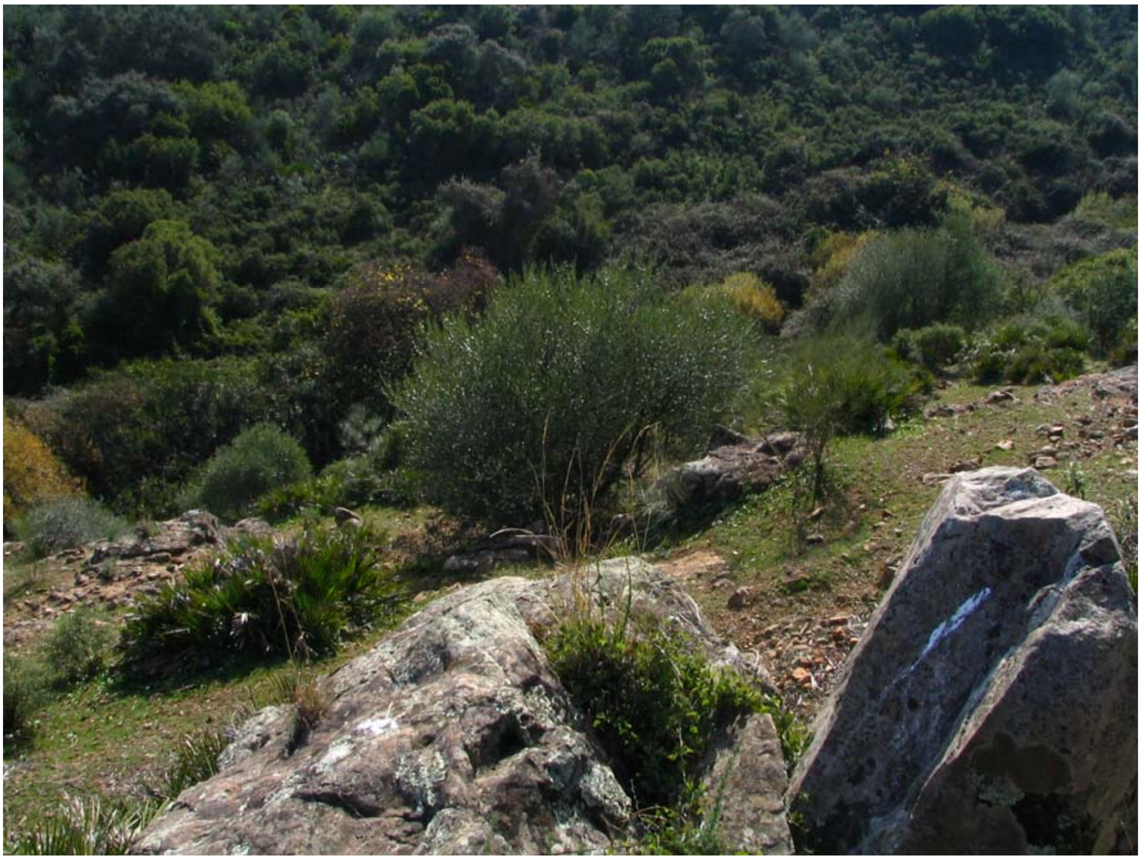

B
