# Supplementary material for: Owls May Use Faeces and Prey Feathers to Signal Current Reproduction
Source: PLoS One. 2008 Aug 20;3(8):e3014. doi: 10.1371/journal.pone.0003014 (PMC2507733; doi:10.1371/journal.pone.0003014)
Supplement: Figure S10 — In the absence of dominant posts, eagle owls use different locations to signal their breeding status, such as trunks, fences, poles and human structures. Faecal marks and plucking sites could also function as visual signals in other avian species, such as the Little Owl, Athene noctua. (0.29 MB PDF) [file pone.0003014.s010.pdf]

S10: ADDITIONAL MARKING POSTS AND OTHER MARKING BIRD SPECIES

In the absence of dominant posts (e.g. in flatlands, marshes, woodlands), eagle owls use different locations to signal their breeding status, such as trunks (e.g. between two owl territories - A and B), fences, poles (C and D), or human structures (E).

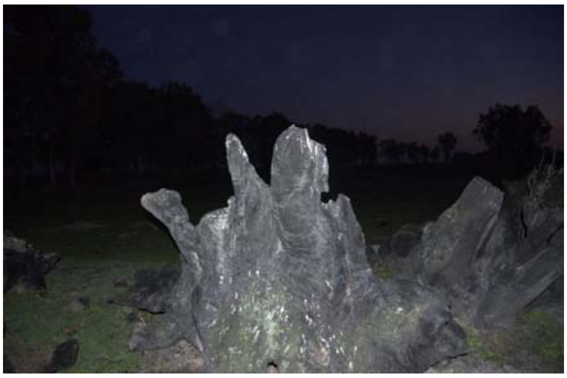

A

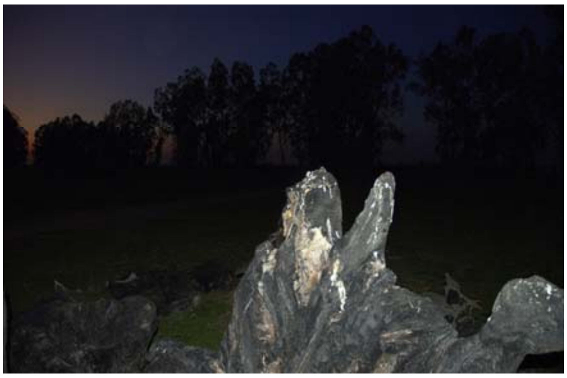

B

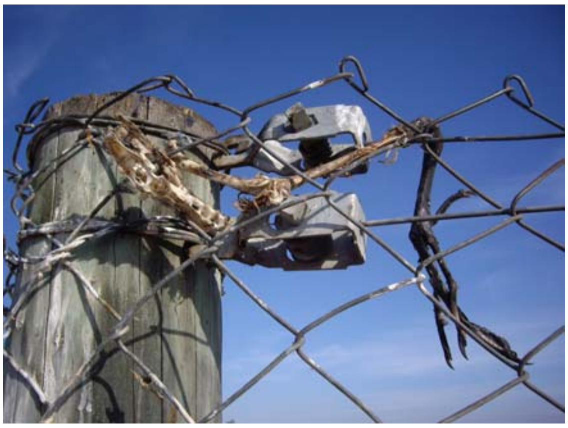

C

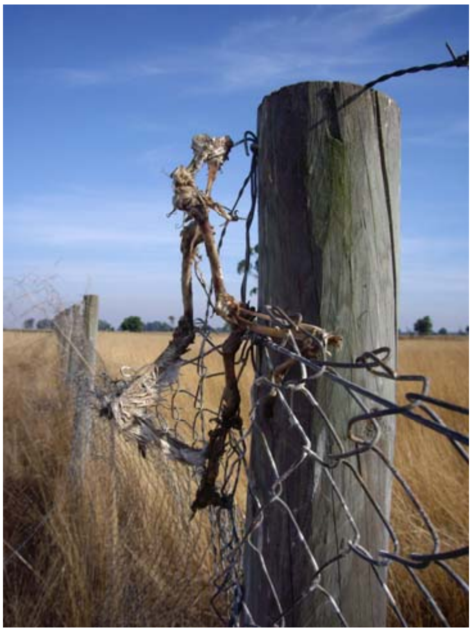

D

S10: ADDITIONAL MARKING POSTS AND OTHER MARKING BIRD SPECIES

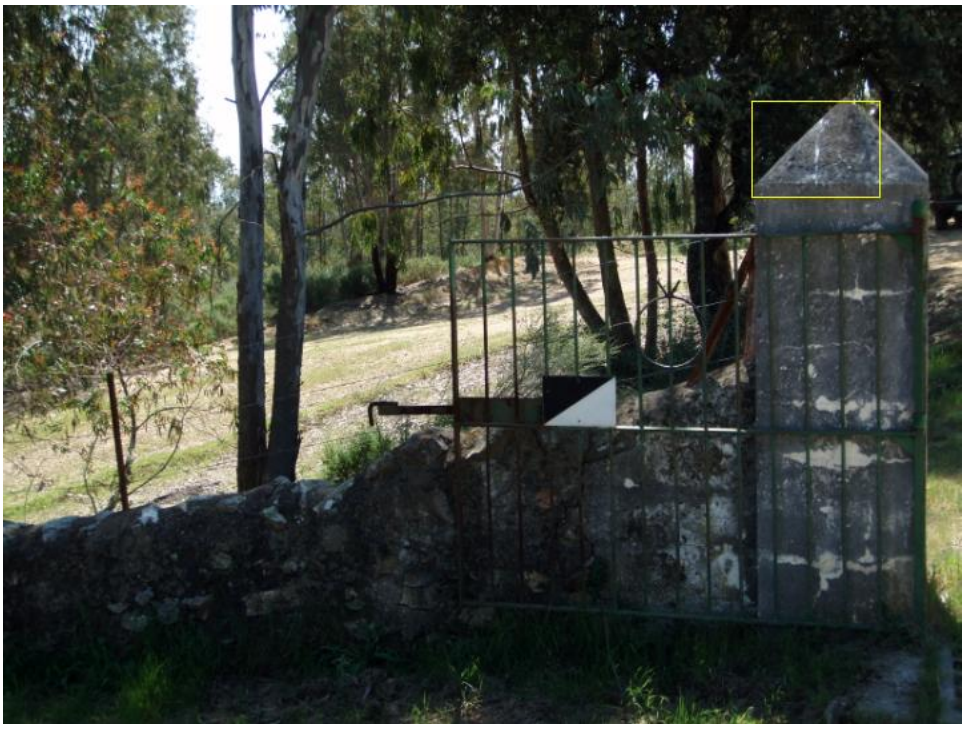

E

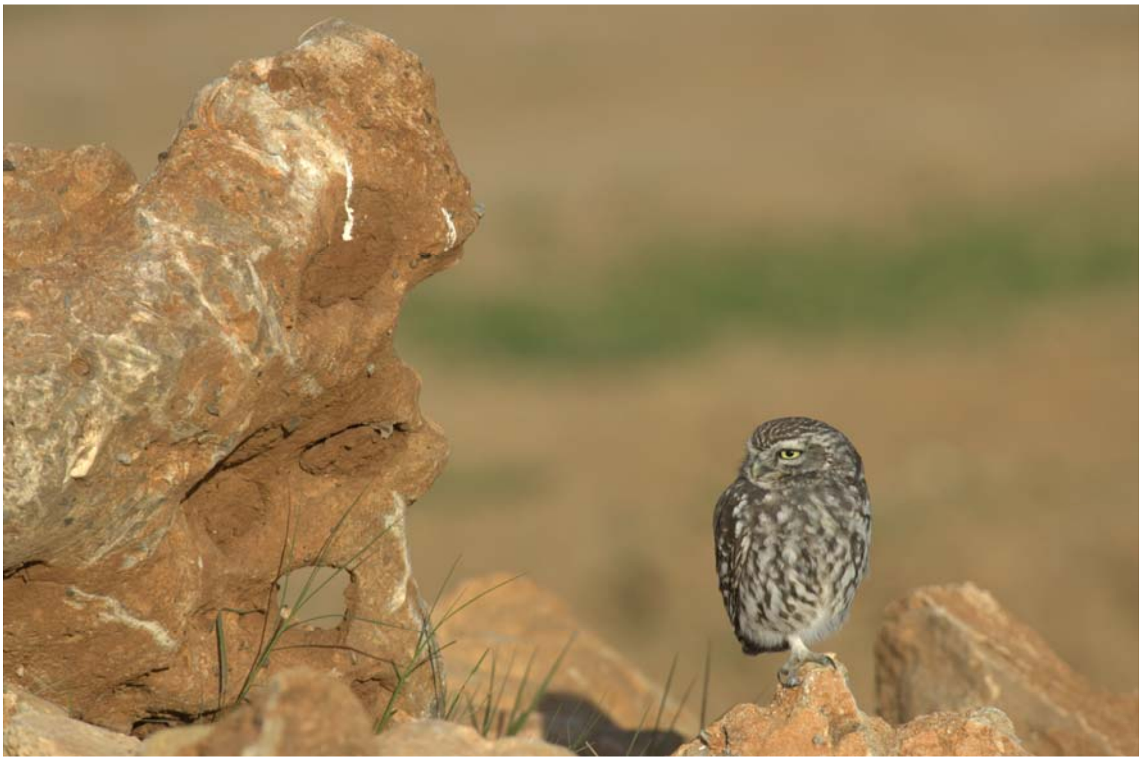

F

Faecal marks and plucking sites could also function as overlooked visual signals in other avian species. For example, white faeces are frequently seen in highly visible positions near the nests of the Little Owl, *Athene noctua* (F).
